# RIOK2 phosphorylation by RSK promotes synthesis of the human small ribosomal subunit

**DOI:** 10.1371/journal.pgen.1009583

**Published:** 2021-06-14

**Authors:** Emilie L. Cerezo, Thibault Houles, Oriane Lié, Marie-Kerguelen Sarthou, Charlotte Audoynaud, Geneviève Lavoie, Maral Halladjian, Sylvain Cantaloube, Carine Froment, Odile Burlet-Schiltz, Yves Henry, Philippe P. Roux, Anthony K. Henras, Yves Romeo

**Affiliations:** 1 Molecular, Cellular and Developmental biology department (MCD), Centre de Biologie Intégrative (CBI), Université de Toulouse, CNRS, UPS, Toulouse, France; 2 Institute for Research in Immunology and Cancer, Université de Montréal, Montreal, Québec, Canada; 3 Institut de Pharmacologie et Biologie Structurale (IPBS), Université de Toulouse, UPS, CNRS, Toulouse, France; 4 Department of Pathology and Cell Biology, Faculty of Medicine, Université de Montréal, Montreal, Québec, Canada; HudsonAlpha Institute for Biotechnology, UNITED STATES

## Abstract

Ribosome biogenesis lies at the nexus of various signaling pathways coordinating protein synthesis with cell growth and proliferation. This process is regulated by well-described transcriptional mechanisms, but a growing body of evidence indicates that other levels of regulation exist. Here we show that the Ras/mitogen-activated protein kinase (MAPK) pathway stimulates post-transcriptional stages of human ribosome synthesis. We identify RIOK2, a pre-40S particle assembly factor, as a new target of the MAPK-activated kinase RSK. RIOK2 phosphorylation by RSK stimulates cytoplasmic maturation of late pre-40S particles, which is required for optimal protein synthesis and cell proliferation. RIOK2 phosphorylation facilitates its release from pre-40S particles and its nuclear re-import, prior to completion of small ribosomal subunits. Our results bring a detailed mechanistic link between the Ras/MAPK pathway and the maturation of human pre-40S particles, which opens a hitherto poorly explored area of ribosome biogenesis.

## Author summary

Ribosomes are universal molecular machines composed of ribosomal RNAs (rRNAs) and ribosomal proteins (RPs) responsible for the synthesis of cellular proteins. In eukaryotic cells, ribosome biogenesis relies on the assembly of large precursor particles (pre-ribosomes) containing, in addition to rRNAs and RPs, more than 200 accessory proteins named assembly and maturation factors (AMFs). Ribosome biogenesis is a highly energy demanding process which is tightly regulated to accommodate protein synthesis with cell growth requirements. In humans, the Ras/MAPK regulatory pathway is one of the main systems controlling cell growth and proliferation. Under favorable growth conditions, the Ras/MAPK pathway activates several aspects of ribosome production, such as synthesis of rRNAs and RPs. Our study highlights that the MAPK pathway also directly stimulates the assembly and maturation of pre-ribosomes. We identify the kinase RIOK2, an AMF involved in the synthesis of the small (40S) ribosomal subunit, as a new target of the MAPK-activated kinase RSK. We describe molecular events promoted by the MAPK pathway to finely tune the activity of RIOK2 during pre-40S particle maturation. This work is particularly important because of the current lack of mechanistic connections between growth-promoting signaling pathways and the assembly and maturation of pre-ribosomes in human cells.

## Introduction

Ribosomes are the universal macromolecular machines responsible for protein synthesis. Eukaryotic ribosomes consist of two subunits (40S and 60S) containing four ribosomal RNAs (rRNAs) and approximately 80 ribosomal proteins (RPs). Ribosome biogenesis begins in the nucleolus with the synthesis by RNA polymerase I (Pol I) of a polycistronic transcript precursor to the 18S, 5.8S and 28S rRNAs and of a precursor to the 5S rRNA by Pol III (S1A Fig). The nascent Pol I transcript is co-transcriptionally packaged into a pre-ribosomal particle that undergoes a series of maturation steps comprising folding and nucleolytic processing of the precursor RNA, chemical modifications of selected nucleotides, and incorporation of RPs and the 5S ribonucleoprotein particle (RNP). Early in the pathway, two discrete particles are generated, the pre-40S and pre-60S particles, precursors to the small and large subunits, respectively. These particles undergo independent maturation steps in the nucleolus and nucleoplasm, before being exported through the nuclear pore complexes. Once in the cytoplasm, pre-40S and pre-60S particles undergo final maturation steps before entering the pool of translation-competent subunits [1,2].

Assembly and maturation of pre-ribosomes is promoted by scores of accessory factors associating transiently with the precursor particles and collectively referred to as assembly and maturation factors (AMFs) [1–4]. Some of these factors participate in the structuring of pre-ribosomes via RNA-binding and/or protein-protein interaction domains, whereas others carry different enzymatic activities, such as nucleases, nucleotide modifying enzymes, putative RNA helicases, kinases, ATPases or GTPases. Ribosome AMFs have been rather exhaustively identified in yeast and the function of some of them is quite well characterized [5]. In human cells, ribosome synthesis appears more complex since large-scale studies revealed that it mobilizes an increased number of AMFs [6–9] and pre-rRNA processing involves a more complex series of events compared to yeast [1,10].

In yeast, ribosome biogenesis is one of the most energetically demanding cellular activities [11,12]. This process also represents a substantial expenditure of energy in human cells since growing HeLa cells produce around 7500 ribosomal subunits per minute [13]. This process needs to be tightly and dynamically regulated to accommodate cell growth and proliferation under favorable conditions, and to prevent energy waste under limiting conditions. The signal transduction cascades Ras/mitogen-activated protein kinase (MAPK) and phosphoinositide 3-kinase (PI3K)/AKT pathways activate ribosome biogenesis in response to external growth factors, mitogens and hormones or changes in intracellular nutrients [14–17]. Upon activation of the Ras/MAPK pathway (S1B Fig), the MAPKs ERK1/2 phosphorylate scores of substrates [18], including members of the RSK (p90 ribosomal S6 kinase) family of protein kinases [19,20], which collectively promote cell growth and proliferation. The PI3K/AKT signaling pathway activates the mechanistic target of rapamycin (mTOR), a conserved Ser/Thr kinase that is found within two different complexes, referred to as mTORC1 and mTORC2. They are distinguished by their accessory proteins, their differential sensitivity to rapamycin inhibitor, and their largely non-overlapping substrates [21].

Sustained activation of both MAPK and mTORC1 pathways increases global translation to feed the protein needs under conditions of growth and proliferation. This is achieved in part by promoting ribosome biogenesis, through the regulation of multiple stages of the process (a global view of the function of ERK and RSK kinases in this process is shown in S1A Fig). They stimulate rDNA transcription by RNA polymerases I and III [22]. ERK1/2 and RSK, as well as mTORC1 and its downstream target S6K, phosphorylate major Pol I (RRN3/TIFI-A and UBF) and Pol III (TFIIIB) transcription factors, and thereby increase synthesis of the 47S pre-rRNA (Pol I) and the 5S rRNA (Pol III) [23–30]. In addition, mTORC1 stimulates transcription of genes encoding RPs and AMFs through the activity of S6Ks [31]. Both pathways also promote translation of mRNAs encoding RPs and AMFs, thereby increasing the supply in ribosome components and ribosome biogenesis factors [32–36]. Especially, mTORC1 regulates translation of a subset of mRNAs possessing a 5′ terminal oligopyrimidine (TOP) motif, which encode components of the translation apparatus, including RPs [37].

By co-activating the syntheses of rRNAs, RPs and AMFs, MAPK and mTORC1 pathways participate in stoichiometric expression of ribosome components and ribosome synthesis machinery, and thereby coordinate the initial stages of ribosome production. Evidence in the literature suggests that these two pathways also regulate the post-transcriptional steps of ribosome synthesis. MAPK and mTORC1 pathways seem to promote pre-rRNA processing [38,39], and rapamycin treatment induces the mislocalisation of several AMFs both in yeast and human cells [40–43]. Although these data suggest that pre-ribosome assembly and maturation are also under the control of signaling pathways, no search has been undertaken so far to identify direct targets of the MAPK and mTORC1 signaling pathways among the large number of pre-ribosome AMFs.

Here we report that the MAPK pathway directly regulates discrete molecular events during the post-transcriptional steps of ribosome biogenesis, which fills a major gap between currently known functions of MAPK signaling in Pol I transcription and cytoplasmic translation. Our results unravel a link between RSK signaling and the maturation of human pre-40S particles, through the regulation of RIOK2, an atypical protein kinase of the RIO family involved in the synthesis of the small ribosomal subunit [44].

## Results

### The MAPK pathway regulates post-transcriptional stages of ribosome biogenesis

**Fig 1:**
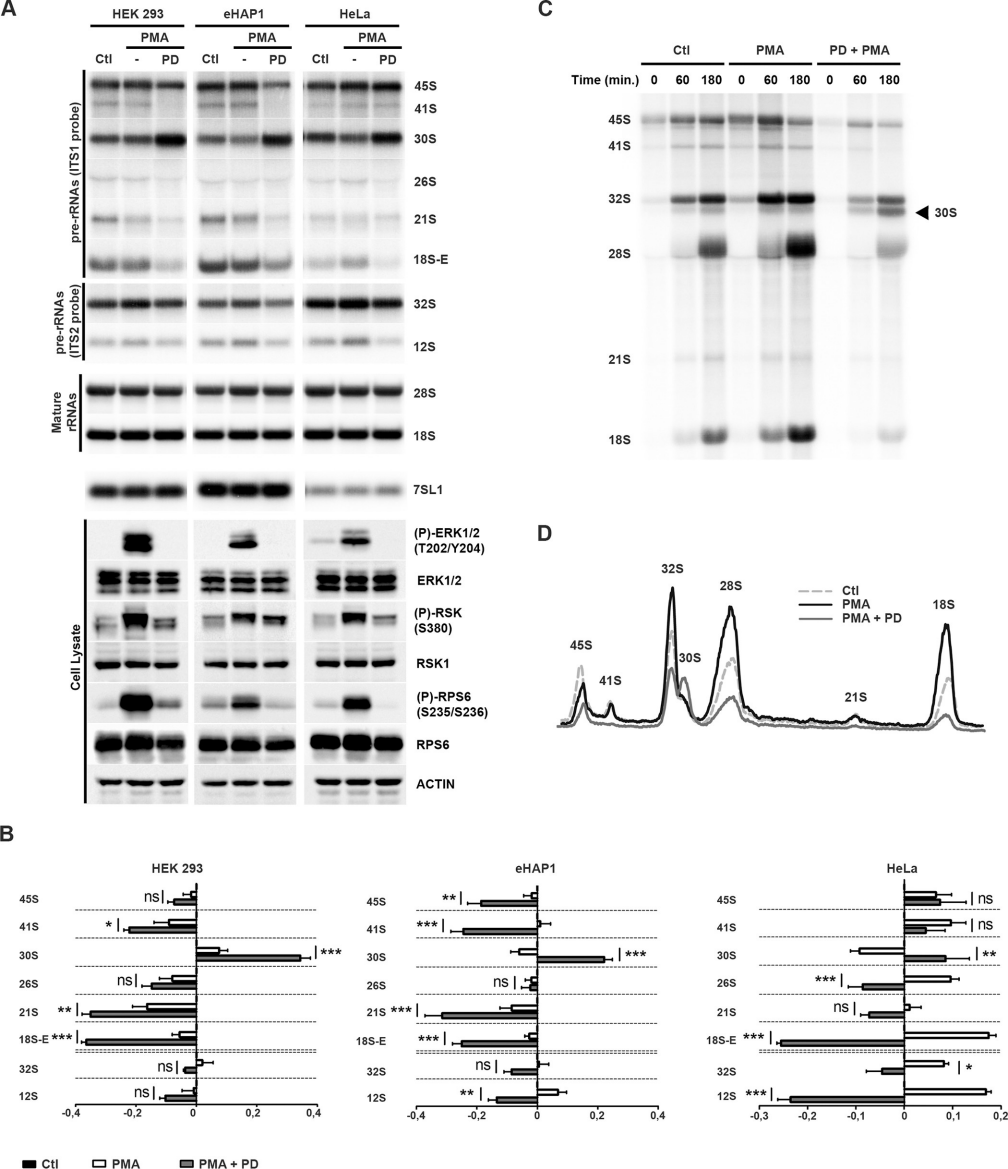
The MAPK signaling pathway regulates post-transcriptional stages of ribosome biogenesis. *(A)* Serum-starved HEK293, eHAP1 or HeLa cells were stimulated (PMA) or not (Ctl) with PMA (100 ng/ml) for 30 min, then treated (PD) or not (-) with 10 μM of PD184352 for 3h. Total RNAs were extracted and the levels of precursor and mature rRNAs and 7SL1 RNA were monitored by Northern blotting (NB, see S1A Fig for a detailed representation of the human pre-rRNA processing pathway and position of the probes). Total proteins were extracted and analyzed by Western blotting (WB) using the indicated antibodies. *(B)* RAMP analyses of pre-rRNA levels obtained in *(A)* normalized to the 7SL1 signals after quantification using MultiGauge software (Fujifilm). Graphical representations show fold changes compared to the starved conditions (Ctl). Statistically significant differences are indicated by asterisks (***: P≤0.001, **: P≤0.01, *: P≤0.05, Two-way ANOVA, Bonferroni posttests). *(C)* Serum-starved (Ctl) HEK293 cells were treated (PD+PMA) or not (PMA) with MEK 1/2 inhibitor PD184352 prior to PMA stimulation. After a 1 h phosphate deprivation, neo-synthesized RNAs were labeled for 1 h with ^32^P-labeled orthophosphate. Cells were harvested 0, 60 and 180 min following addition of cold orthophosphate. Total RNAs were extracted, separated by electrophoresis and transferred to nylon membranes. The labeled (pre-)rRNAs indicated on the left and on the right were detected by autoradiography. *(D)* Graphical representation of radioactive signals obtained in *(C)* after quantification using MultiGauge software (Fujifilm).

To assess whether the MAPK signaling pathway regulates pre-rRNA processing in human cells, we first examined the levels of various rRNA precursors to both the small and large ribosomal subunits (maturation pathway depicted in S1A Fig) upon pharmacological inhibition of ERK1/2 kinases in three different human cell lines (HEK293, eHAP1 and HeLa). Cells were serum-starved to reduce signaling pathways to basal levels and incubated with PMA (phorbol 12-myristate 13-acetate) to stimulate the MAPK pathway prior to treatment with MEK1/2 (PD184352) inhibitor (S1B Fig). In this and all subsequent experiments, we have performed western blotting to assess the activation levels of ERK1/2 and RSK kinases (Fig 1A, lower panels). Efficient activation of the MAPK pathway by PMA, serum or epidermal growth factor (EGF) was shown using phospho-specific antibodies targeted against phosphorylated ERK1/2 (T202/Y204) and RSK (S380). Using these tools, we found that treatment with MEK1/2 inhibitor (PD184352) abrogated ERK1/2 and RSK phosphorylation. The inhibitor efficiency was also attested by the strong decrease in RPS6 phosphorylation at Ser235/236, which is known to be regulated by RSK [45]. In all cell lines, treatments with MEK1/2 inhibitor induced changes in the processing pathways leading to the production of both the 18S and 28S rRNAs. In the small subunit biogenesis pathway, inhibition of MEK1/2 induced a strong accumulation of the 30S precursor and a marked reduction in the production of all downstream intermediates, in particular the 18S-E pre-rRNA, which is the ultimate precursor to the mature 18S rRNA (Fig 1A, ITS1 probe). In this and all subsequent Northern blotting experiments, the relative abundance of the pre-rRNA species was quantified using the “Ratio Analysis of Multiple Precursors” (RAMP) procedure [46] (Fig 1B). With regards to the large ribosomal subunit processing pathway, production of the 32S and 12S precursors was affected to some extent by MAPK pathway inhibition (Fig 1A, ITS2 probe and RAMP quantifications in Fig 1B). These profiles indicate that inhibition of MAPK signaling induces changes in the steady-state levels of several intermediates in the maturation pathways leading to both ribosomal subunits.

Northern blotting experiments did not show any drastic change in mature 18S and 28S rRNA steady-state levels in these cell lines (Fig 1A, mature rRNAs, and RAMP quantifications in S1C Fig). To get a more dynamic assessment of the role of the MAPK signaling pathway in rRNA production, we performed pulse-chase analyses of rRNA synthesis using [^32^P]-ortho-phosphate. Treatment of serum-starved HEK293 cells with PMA increased the levels of the 45S pre-rRNA, consistent with an activation of Pol I transcription. All downstream rRNA precursors of both pre-40S and pre-60S maturation pathways also accumulated to higher levels upon PMA treatment, and consequently, production of the mature 18S and 28S rRNAs was remarkably increased (Fig 1C and 1D). Inhibition of MEK1/2 induced a marked delay in the production of both the 18S and 28S rRNAs. Importantly, and consistent with the Northern blotting experiments presented in Fig 1A, cells treated with MEK1/2 inhibitors (PD184352) displayed a pronounced accumulation of the early 30S precursor to the 18S rRNA, which was barely detected in PMA-treated cells, indicating that the maturation of this precursor is affected upon inhibition of the MAPK pathway. Altogether, these data show that the MAPK pathway regulates both the synthesis of the 45S pre-rRNA and subsequent maturation events leading to the biogenesis of both ribosomal subunits in human cells.

### RIOK2 is phosphorylated at Ser483 upon activation of the MAPK pathway

RSK is the most prominent effector kinase that operates downstream of ERK1/2, suggesting that RSK could regulate post-transcriptional steps of ribosome synthesis, possibly by modulating the activity of selected pre-ribosome AMFs. To address this hypothesis, we used the Scansite bioinformatics tool (https://scansite4.mit.edu/4.0/) to search for the canonical Arg/Lys-X-Arg/Lys-X-X-pSer/Thr (RXRXXpS/T) motif found in RSK substrates, within the sequences of an exhaustive list of human AMFs [5,7,8]. We found that the 552 amino acid protein kinase RIOK2 features high confidence RXRXXpS/T phosphorylation motifs. Numerous phosphorylated sites have been detected in RIOK2 (https://www.phosphosite.org/proteinAction.action?id=2360&showAllSites=true) but the protein only contains two predicted RXRXXpS/T sites in its C-terminal region: a high-stringency site predicting phosphorylation at Ser483 (S483) and a medium-stringency site at Thr481 (T481) (S2A Fig).

**Fig 2:**
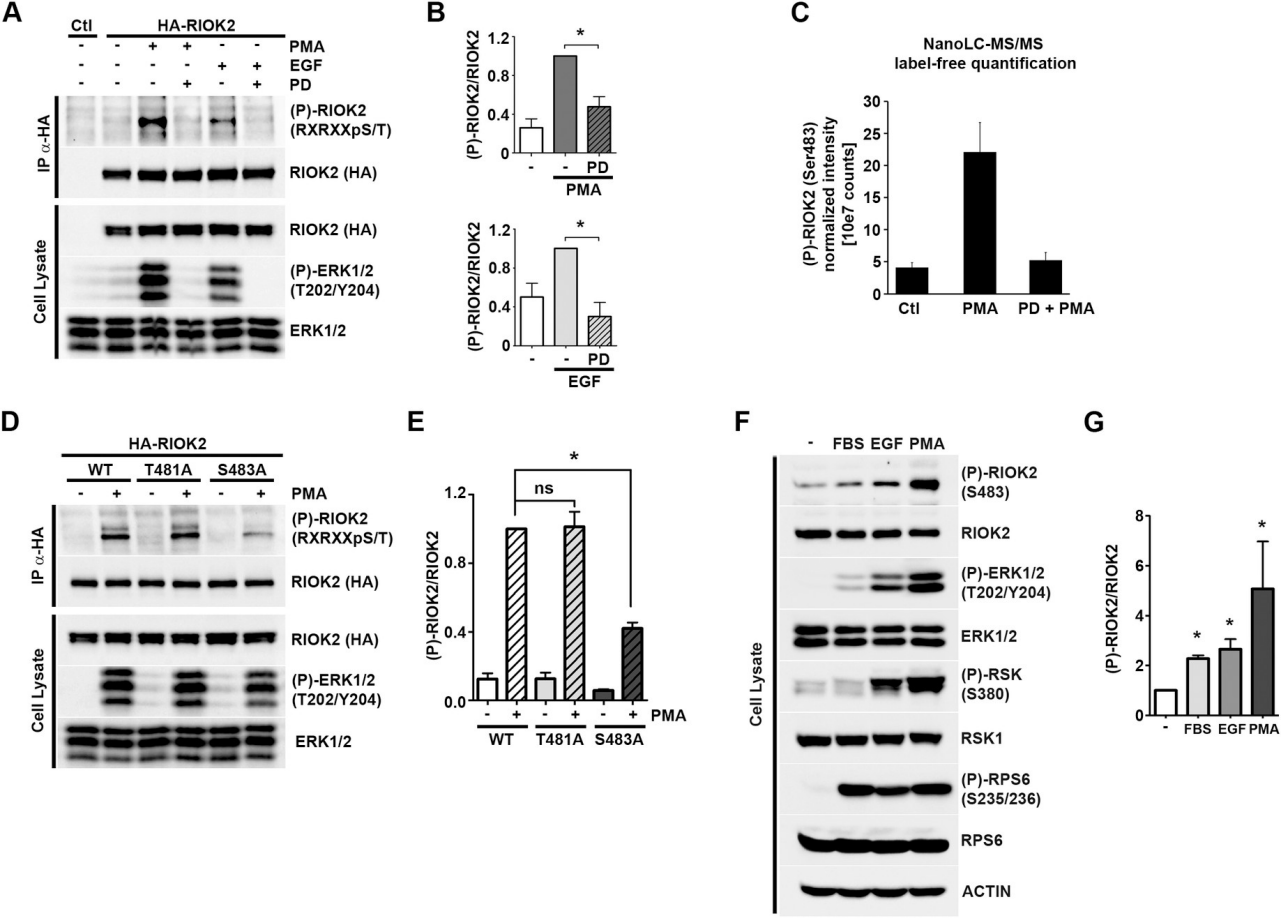
RIOK2 is phosphorylated at Ser483 upon activation of the MAPK pathway. *(A)* HEK293 cells were transfected with a plasmid expressing HA-RIOK2 or an empty vector (Ctl). HA-RIOK2 was immunoprecipitated from serum-starved cells treated (+) or not (-) with PD184352 for 1h (10 μM) prior to PMA (100 ng/ml, 20 min) or EGF (25 μg/ml, 10 min) stimulation (+). Samples were analyzed by WB using anti-RXRXXpS/T or anti-HA antibodies. *(B)* RXRXXpS/T ((P)-RIOK2) and total RIOK2 signals obtained in *(A)* were quantified using ImageLab software and expressed as fold change relative to the PMA condition. Statistically significant differences are indicated by asterisks (*: P≤0.05, One-tailed Mann Whitney test). *(C)* HA-RIOK2 was immunoprecipitated from serum-starved (Ctl) HEK293 cells treated (PMA+PD) or not (PMA) with PD184352 (10 μM, 1h) prior to PMA stimulation (100 ng/ml, 20 min). Purified HA-RIOK2 was isolated following SDS-PAGE, in gel digested with trypsin and the resulting peptides were submitted to nano-LC-MS/MS analysis. Label-free quantitative analysis of phosphorylation of the different RIOK2 phospho-peptides was performed as specified in the Materials and Methods section. Data are representative of triple biological replicate experiments for each condition. *(D)* HEK293 cells expressing HA-tagged versions of WT or mutant versions of RIOK2 (T481A or S483A) were serum starved (-) prior to PMA (100 ng/ml, 20 min) stimulation (+). HA-RIOK2 was immunoprecipitated and samples were analyzed by WB as in *(A)*. *(E)* RXRXXpS/T ((P)-RIOK2) and total RIOK2 signals obtained in *(D)* were quantified using ImageLab software and expressed as fold change relative to the WT + PMA condition. Statistically significant differences are indicated by asterisks (*: P≤0.05, One-tailed Mann Whitney test). *(F)* Serum-starved HEK293 cells were stimulated with different agonists of the MAPK pathway. Phosphorylation of endogenous RIOK2 at Ser483 ((P)-RIOK2) was monitored by WB using specific antibodies. *(G)* (P)-RIOK2 and total RIOK2 signals obtained in *(F)* were quantified using ImageLab software and expressed as fold change relative to the starved condition. Statistically significant differences are indicated by asterisks (*: P≤0.05, One-tailed Mann Whitney test).

To determine whether RIOK2 is phosphorylated at RXRXXpS/T motifs, endogenous RIOK2 was immunoprecipitated from serum-growing HEK293 cells and its phosphorylation was analyzed by immunoblotting using antibodies detecting the phosphorylated consensus RXRXXpS/T motif (S2B Fig). We observed that RIOK2 was indeed phosphorylated at one or several RXRXXpS/T motif(s) and that the signal disappeared upon treatment of the immunoprecipitate by λ phosphatase, attesting for the presence of a phosphate moiety. To determine whether this phosphorylation event responds to MAPK pathway activation, HEK293 cells transiently expressing HA-tagged RIOK2 were serum-starved and stimulated with EGF or PMA agonists, and phosphorylation of immunoprecipitated HA-RIOK2 was analyzed by immunoblotting (Fig 2A and quantifications in Fig 2B). Notably, we found that activation of the MAPK pathway stimulated RIOK2 phosphorylation at the RXRXXpS/T motif. Pre-treatment of starved cells with MEK1/2 inhibitor (PD184352) abrogated the induction of RIOK2 phosphorylation in response to EGF or PMA, indicating that RIOK2 is phosphorylated on RXRXXpS/T consensus sites in a MAPK-dependent manner.

We next attempted to identify RIOK2 phosphorylation sites that are regulated by MAPK signaling using a label-free quantitative mass spectrometry (MS) approach. HEK293 cells expressing HA-tagged RIOK2 were serum-starved overnight and pre-treated or not with MEK1/2 inhibitor (PD184352) prior to PMA stimulation (S2C Fig). Immunoprecipitated HA-RIOK2 was isolated using SDS-PAGE and digested in-gel with trypsin. Samples were analyzed by nano-liquid chromatography-tandem MS (nanoLC-MS/MS) to detect the presence of putative phosphorylation sites. We evaluated the relative abundance of all phosphopeptides identified in our study, corresponding to 12 phosphorylation sites (S1 Table). Among these, Ser483 was the only RIOK2 residue whose phosphorylation robustly increased upon PMA stimulation and returned to basal levels upon MEK1/2 inhibition (Figs 2C and S2D). Phosphorylation of the other predicted RXRXXpS/T phosphorylation site (Thr481) was not detected, suggesting that this residue is not phosphorylated in HEK293 cells. Altogether, these results suggest that Ser483 is the only residue of RIOK2 whose phosphorylation is under the control of MAPK signaling. Interestingly, the RSK phosphorylation motif containing Ser483 is conserved within vertebrates, suggesting that it is involved in an important biological function (S2E Fig).

To further confirm that the MAPK pathway induces RIOK2 phosphorylation at Ser483, we transiently expressed in HEK293 cells HA-tagged versions of WT or mutant RIOK2 in which Ser483 or Thr481 were substituted for a non-phosphorylatable alanine (RIOK2^S483A^, RIOK2^T481A^) (Fig 2D). These cells were serum-starved, stimulated with PMA, and HA-RIOK2 was purified from cell lysates and analyzed by immunoblotting. Mutation of Ser483 almost completely prevented RIOK2 phosphorylation after PMA stimulation. Quantifications of phosphorylation signals (Fig 2E) showed a residual phosphorylation of RIOK2^S483A^ that may be due to other basophilic kinases, which phosphorylate Ser/Thr in the vicinity of Arg residues, or compensatory phosphorylation effects at sites not normally recognized by RSK. Since mutation of Thr481 alone did not reduce RIOK2 phosphorylation and since we did not detect Thr481 phosphorylation *in vivo*, we concluded that activation of MAPK signaling induces RIOK2 phosphorylation specifically at Ser483 and we therefore focused solely on this site in the functional study described below.

To validate these results, we generated antibodies specifically directed against the Ser483-phosphorylated RIOK2 peptide and monitored the phosphorylation status of endogenous RIOK2 in serum-starved HEK293 cells or in response to agonists of the MAPK pathway (Fig 2F and quantifications in Fig 2G). Phosphorylation of RIOK2 at Ser483 increased upon stimulation with serum, EGF or PMA, indicating that activation of MAPK signaling results in phosphorylation of endogenous RIOK2 at Ser483. In addition, RIOK2 phosphorylation levels correlated well with those of ERK and RSK kinases, suggesting a direct regulation of RIOK2 by at least one of these kinases.

### RIOK2 is a direct RSK substrate

**Fig 3:**
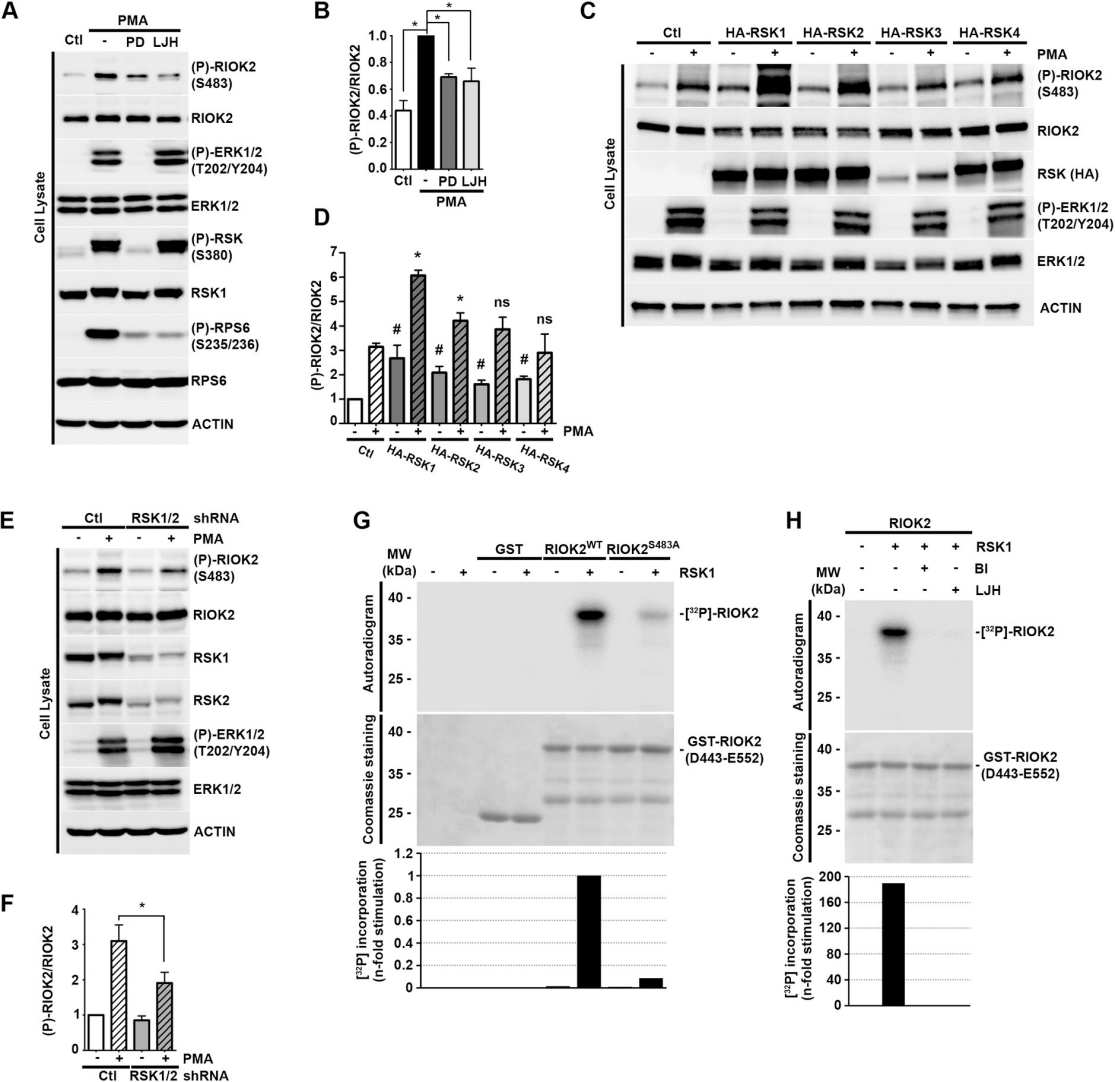
RIOK2 is a direct RSK substrate. *(A)* Serum-starved (Ctl) HEK293 cells were treated or not (-) with PD184352 (PD), or LJH685 (10 μM) (LJH) for 1 h prior to PMA stimulation (100 ng/ml, 20 min). Phosphorylation of endogenous RIOK2 at Ser483 ((P)-RIOK2) was monitored by WB using specific antibodies. *(B)* (P)-RIOK2 and total RIOK2 signals obtained in *(A)* were quantified using ImageLab software and expressed as fold change relative to the PMA condition. Statistically significant differences are indicated by asterisks (*: P≤0.05, One-tailed Mann Whitney test). *(C)* HEK293 cells were transfected with vectors over-expressing HA-tagged RSK1, RSK2, RSK3 or RSK4, or the empty vector (Ctl). Following serum-starvation and PMA stimulation (100 ng/ml, 20 min), samples were analyzed by WB using the indicated antibodies. *(D)* (P)-RIOK2 and total RIOK2 signals obtained in *(C)* were quantified using ImageLab software and expressed as fold change relative to starved Ctl cells. Statistically significant differences between starved conditions relative to starved Ctl cells are indicated by hashes, and between PMA stimulated conditions relative to stimulated Ctl cells by asterisks (#/*: P≤0.05, ns: not statistically significant, One-tailed Mann Whitney test). *(E)* HEK293 cells expressing shRNA targeting an irrelevant sequence (Ctl) or both RSK1 and RSK2 (RSK1/2) were processed as in *(C)*. *(F)* (P)-RIOK2 and total RIOK2 signals obtained in *(E)* were quantified using ImageLab software and expressed as fold change relative to the starved condition. Statistically significant differences are indicated by asterisks (*: P≤0.05, One-tailed Mann Whitney test). *(G)* Human activated RSK1 was incubated in the presence of γ[^32^P]-ATP with either GST alone, or a GST-RIOK2 peptide (D443-E552) containing either S483 (RIOK2^WT^) or the non-phosphorylatable version (RIOK2^S483A^). The resulting samples were analyzed by SDS-PAGE and revealed by autoradiography or Coomassie blue staining. Quantification of [^32^P] incorporation within each peptide is expressed as n-fold change compared to the absence of RSK1. *(H)* In vitro kinase assays performed as in *(G)* in the presence or not of RSK inhibitors BI-D1870 or LJH685 (10 mM).

To test whether RSK is responsible for RIOK2 phosphorylation at Ser483, we treated serum-starved HEK293 cells with PMA, with or without prior treatment with the MEK1/2 (PD184352) or RSK (LJH685) inhibitors and analyzed RIOK2 phosphorylation at S483 (Fig 3A and quantifications in Fig 3B). As LJH685 targets the N-terminal kinase domain of RSK, it does not prevent its phosphorylation at S380 by the C-terminal kinase domain. As in Fig 1, the efficiency of the inhibitors was attested by the strong decrease in RPS6 phosphorylation at Ser235/236. Consistent with a role of RSK in RIOK2 phosphorylation, we observed that treatment of cells with LJH685 inhibitors significantly reduced Ser483 phosphorylation upon PMA stimulation.

The RSK family comprises four closely related Ser/Thr kinases (RSK1-4) expressed from independent genes. Both RSK1 and RSK2 promote cell growth and proliferation, and are the predominant RSK isoforms expressed in HEK293 cells. We next investigated which ones are involved in RIOK2 phosphorylation at Ser483. HEK293 cells over-expressing each of the four RSK isoforms were stimulated with PMA and RIOK2 phosphorylation at Ser483 was analyzed by immunoblotting (Fig 3C and quantifications in Fig 3D). Overexpression of RSK1, and RSK2 to a lesser extent, increased RIOK2 phosphorylation at Ser483 upon PMA treatment. Signal quantifications and associated statistical tests indicated that overexpression of RSK3 or RSK4 did not significantly contribute to RIOK2 phosphorylation. These data suggest that RSK1 and RSK2 are the predominant isoforms involved in the regulation of RIOK2. Of note, we detected lower levels of RSK3 protein in the soluble extract because the protein is largely insoluble when overexpressed, as previously reported [47]. The role of RSK1 and RSK2 was further confirmed through knockdown experiments using stable shRNAs. Consistent with a dual role for RSK1 and RSK2, we found that knockdown of both isoforms resulted in a reduction of RIOK2 Ser483 phosphorylation upon activation of MAPK signaling, compared to control cells (Fig 3E and quantifications in Fig 3F). We noted that RSK knockdown (Fig 3E) or inhibition (Fig 3A) slightly increased ERK phosphorylation levels, most likely due to a previously described negative feedback loop in the MAPK pathway, whereby RSK activation partially inhibits ERK phosphorylation [48–51].

To determine if RIOK2 is a direct RSK substrate, we performed *in vitro* phosphorylation assays using a GST-tagged C-terminal fragment of RIOK2 spanning residues Asp443 to Glu552 purified from *E*. *coli* (Fig 3G and 3H). Upon incubation with active, recombinant human RSK1 produced in insect cells and γ[^32^P]-ATP, this peptide became efficiently phosphorylated (Fig 3G). As specificity controls, we showed that GST alone or a mutant RIOK2 peptide in which Ser483 was replaced by a non-phosphorylatable alanine were poorly phosphorylated by RSK. Addition of ATP-competitive RSK inhibitors (BI-D1870 or LJH685) to the *in vitro* assay compromised [^32^P] incorporation into the RIOK2 peptide, demonstrating that this event requires RSK catalytic activity (Fig 3H). Together, these experiments strongly suggest that RSK directly promotes RIOK2 phosphorylation at Ser483.

### RIOK2 phosphorylation at Ser483 is required for efficient maturation of pre-40S particles

Both yeast Rio2 and human RIOK2 are essential for cell viability [52,53]. In agreement with this, RIOK2 is required for cell proliferation, migration and survival of glioblastoma [54,55]. RIOK2 has been suggested to function in mitotic progression [56] but its best documented molecular function is linked to the synthesis of the small ribosomal subunit. Depletion of Rio2/RIOK2 prevents processing of the last precursor to the mature 18S rRNA (20S pre-rRNA in yeast or 18S-E pre-rRNA in human) within cytoplasmic pre-40S particles, and therefore inhibits production of the 40S subunit [52,57,58]. The precise function of RIOK2 in the maturation of pre-40S particles remains ill-defined. Yeast Rio2 features autophosphorylation and ATPase activities *in vitro* and has been suggested to function as an ATPase in the maturation process rather than a *bona fide* kinase [59]. In contrast, human RIOK2 was recently shown to phosphorylate DIM1 *in vitro*, which is a component of nuclear pre-40S particles [60]. Human RIOK2 forms catalytically inactive homodimers *in vitro*, suggesting that some aspects of RIOK2 regulation *in vivo* may involve dimerization [61].

**Fig 4:**
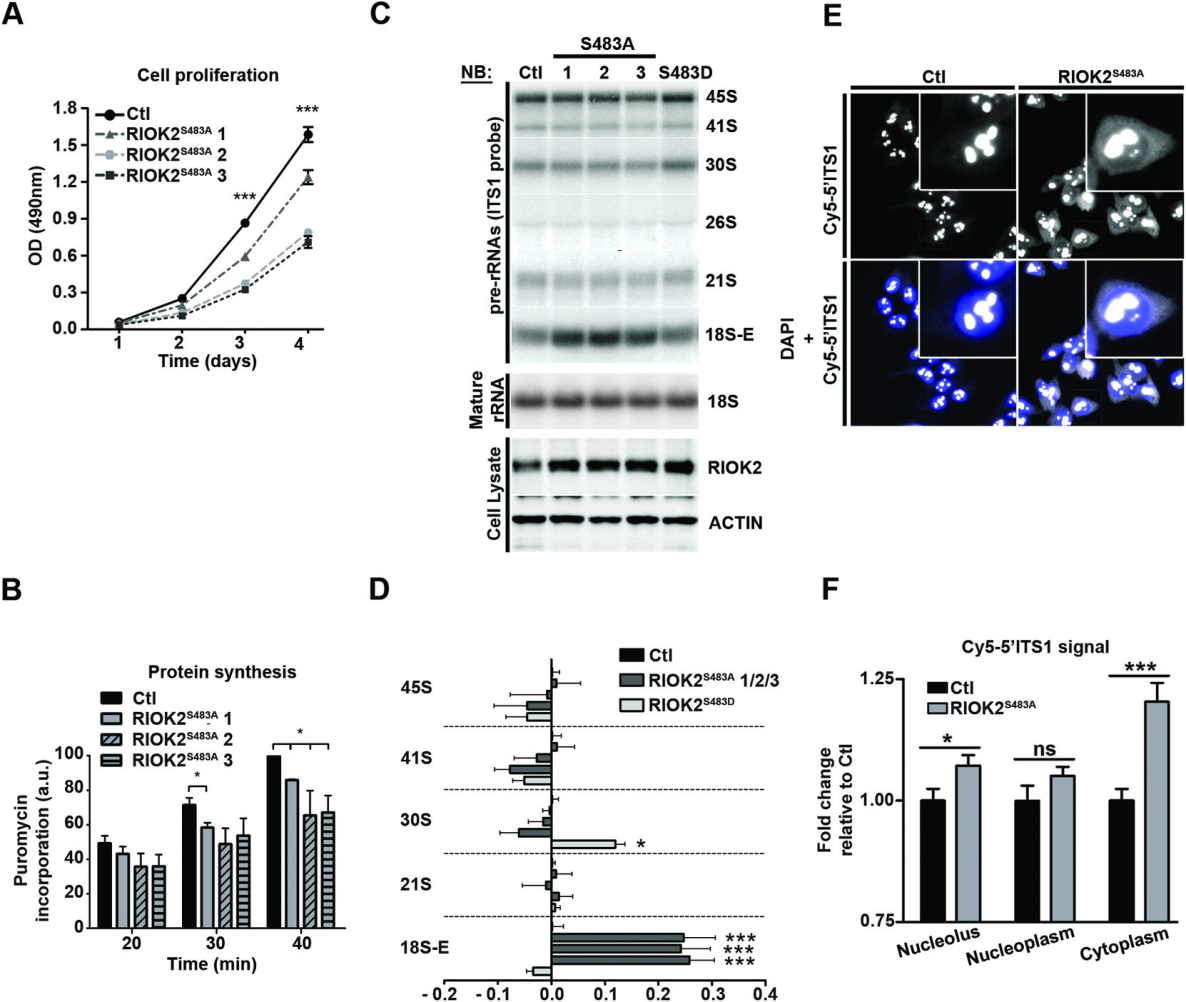
RIOK2 phosphorylation at Ser483 is required for efficient maturation of pre-40S particles. *(A)* MTS assays were performed on Control (Ctl) and RIOK2^S483A^ (1 to 3) eHAP1 cell lines at the indicated time points. ODs at 490 nm were measured using Spectramax. *(B)* Control (Ctl) and RIOK2^S483A^ (1 to 3) eHAP1 cell lines were incubated with 1 μM puromycin for the indicated times. Levels of puromycin-labelled peptides were monitored by WB using anti-puromycin antibodies. WB signals were quantified using ImageLab software and expressed as arbitrary units (a.u.). *(C)*, Total cellular RNAs were extracted from control (Ctl), RIOK2^S483A^ (1 to 3) or RIOK2^S483D^ eHAP1 cell lines. Accumulation levels of pre-rRNAs and mature rRNAs were analyzed by NB as in Fig 1. *(D)* RAMP analyses of pre-rRNA levels obtained in *(C)* normalized to the 26S signals after quantification using MultiGauge software (Fujifilm). Graphical representations show fold changes compared to the control condition (Ctl). Statistically significant differences are indicated by asterisks (***: P≤0.001, *: P≤0.05, Two-way ANOVA, Bonferroni posttests). *(E)* FISH experiments performed on RIOK2^WT^ (Ctl) and RIOK2^S483A^ eHAP1 cell lines. Pre-rRNAs were detected using a Cy5-labeled 5’-ITS1 probe. Cells were stained with DAPI to visualize nuclei, and images were captured in identical setting conditions. *(F)* Nucleolar, nuclear and cytoplasmic fluorescence signals were quantified using ImageJ software, as described in the “Materials and Methods” section and S7 Fig. Graph representations show fold change in RIOK2^S483A^ relative to RIOK2^WT^ cell line (n = 100 cells from different fields). Statistically significant differences are indicated by asterisks (***: P≤0.001, **: P≤0.01, *: P≤0.05, One-tailed Mann Whitney test).

To assess the functional relevance of RIOK2 phosphorylation at Ser483, we used a CRISPR/Cas9-based knock-in approach to generate human eHAP1 haploid cell lines expressing mutant versions of RIOK2, bearing a substitution of Ser483 for either a non-phosphorylatable alanine (RIOK2^S483A^) or a phosphomimetic aspartic acid (RIOK2^S483D^) [62]. Notably, we found that RIOK2^S483A^ mutant cell lines displayed a significantly decreased proliferation rate, as assessed by both MTS assay (Fig 4A) and cell counting (S3A Fig), indicating that phosphorylation of RIOK2 at Ser483 is required for optimal cell proliferation. This proliferation defect was found to be less pronounced than that resulting from treatment with PD184352, LJH685 or BI-D1870 inhibitors (S3B Fig), likely because the latter affect ribosome synthesis at multiple levels (Figs 1 and S1A) and probably also affect several other MAPK-dependent cellular processes. We further observed that the slower proliferation rate of RIOK2^S483A^-expressing mutant cells was not correlated with a significant increase in cell death mechanisms, such as apoptosis (S3C Fig). Interestingly, abrogation of RIOK2 phosphorylation at Ser483 significantly impaired global protein synthesis, as measured using the surface sensing of translation (SUnSET) method [63] (Figs 4B and S3D). Since RIOK2 functions in the last stages of pre-40S particle maturation, these results suggest that RIOK2 phosphorylation at Ser483 is important for production of translation-efficient ribosomes.

To delineate the molecular mechanism at the origin of this phenotype, we analyzed pre-rRNA processing in RIOK2^WT^, RIOK2^S483A^ and RIOK2^S483D^ cell lines. Total RNAs were extracted from these cells and rRNA precursor levels were analyzed by Northern blotting (Fig 4C upper panel and RAMP quantifications in Fig 4D). Interestingly, we found a significant accumulation of the 18S-E precursor in all cell lines expressing RIOK2^S483A^, indicating that the maturation of pre-40S particles is affected by the loss of Ser483 phosphorylation. RIOK2 knockdown in eHAP1 cells resulted in a similar accumulation of 18S-E pre-rRNA (S3E Fig and RAMP quantifications in S3F Fig), suggesting that Ser483 contributes to the regulation of RIOK2 function. Importantly, expression of the RIOK2^S483D^ phospho-mimetic mutant induced distinct processing defects compared to RIOK2^S483A^. Instead of a marked increase in the late 18S-E precursor, we observed on the contrary a slight reduction of this precursor levels in cells expressing RIOK2^S483D^ and an accumulation of the 30S precursor (Fig 4C upper panel and RAMP quantifications in Fig 4D), consistent with processing defects at earlier stages. Since RIOK2 is recruited in nuclear pre-ribosomal particles, these data may suggest that expression of a mutant version of RIOK2 mimicking a constitutive phosphorylation at S483 impairs RIOK2 recruitment into pre-ribosomes. In both cases, no significant change in mature 18S rRNA levels was observed in these cell lines (Figs 4C, middle panel, and S3G and quantifications in S3H Fig), suggesting that the steady state levels of mature 40S ribosomal subunits are not altered. It is worth noting that the S483A and S483D mutations did not decrease the steady-state levels of the mutant RIOK2 proteins (Fig 4C, lower panel), indicating that the processing defects observed in these cell lines do not result from a shortage in cellular RIOK2 protein.

Pre-40S particles containing the 18S-E pre-rRNA are generated in the nucleolus [64]. They undergo maturation steps in the nucleoplasm before being exported to the cytoplasm, where final maturation events lead to the production of the mature 18S rRNA of the 40S subunit [4]. To delineate more precisely which stage of pre-40S particle maturation is delayed in RIOK2^S483A^ cells, we performed fluorescence *in situ* hybridization (FISH) experiments to detect precursors of the 18S rRNA *in situ* in fixed cells. We used a probe detecting all pre-rRNAs of the small subunit maturation pathway (ITS1 probe, S1A Fig) in the nucleoli, nucleoplasm and cytoplasm. FISH signal observed in the cytoplasm corresponds exclusively to the detection of the 18S-E pre-rRNA, the only precursor retaining part of the ITS1 sequence in this compartment. Cells expressing RIOK2^S483A^ displayed a stronger FISH signal in the cytoplasm compared to control cells (Fig 4E and quantifications in Fig 4F), indicating that these cells accumulate the 18S-E precursors, therefore pre-40S particles, in the cytoplasm. Collectively, our results suggest that phosphorylation of RIOK2 at Ser483 facilitates late, cytoplasmic stages of pre-40S particle maturation.

### Loss of RIOK2 phosphorylation at Ser483 increases its association with cytoplasmic pre-40S particles

Rio2/RIOK2 is incorporated into pre-40S particles in the nucleus, participates to their export through direct binding to the CRM1 exportin, and dissociates from cytoplasmic pre-40S particles to get recycled back into the nucleus [52,57,65]. Rio2/RIOK2 catalytic activity contributes to its recycling into the nucleus [57,59,66,67]. Furthermore, a defect in RIOK2 release is correlated with aberrant retention within pre-40S particles of other late AMFs, such as ENP1/Bystin, PNO1/DIM2, LTV1 and NOB1, the endonuclease responsible for conversion of the 18S-E precursor into mature 18S rRNA [57].

**Fig 5:**
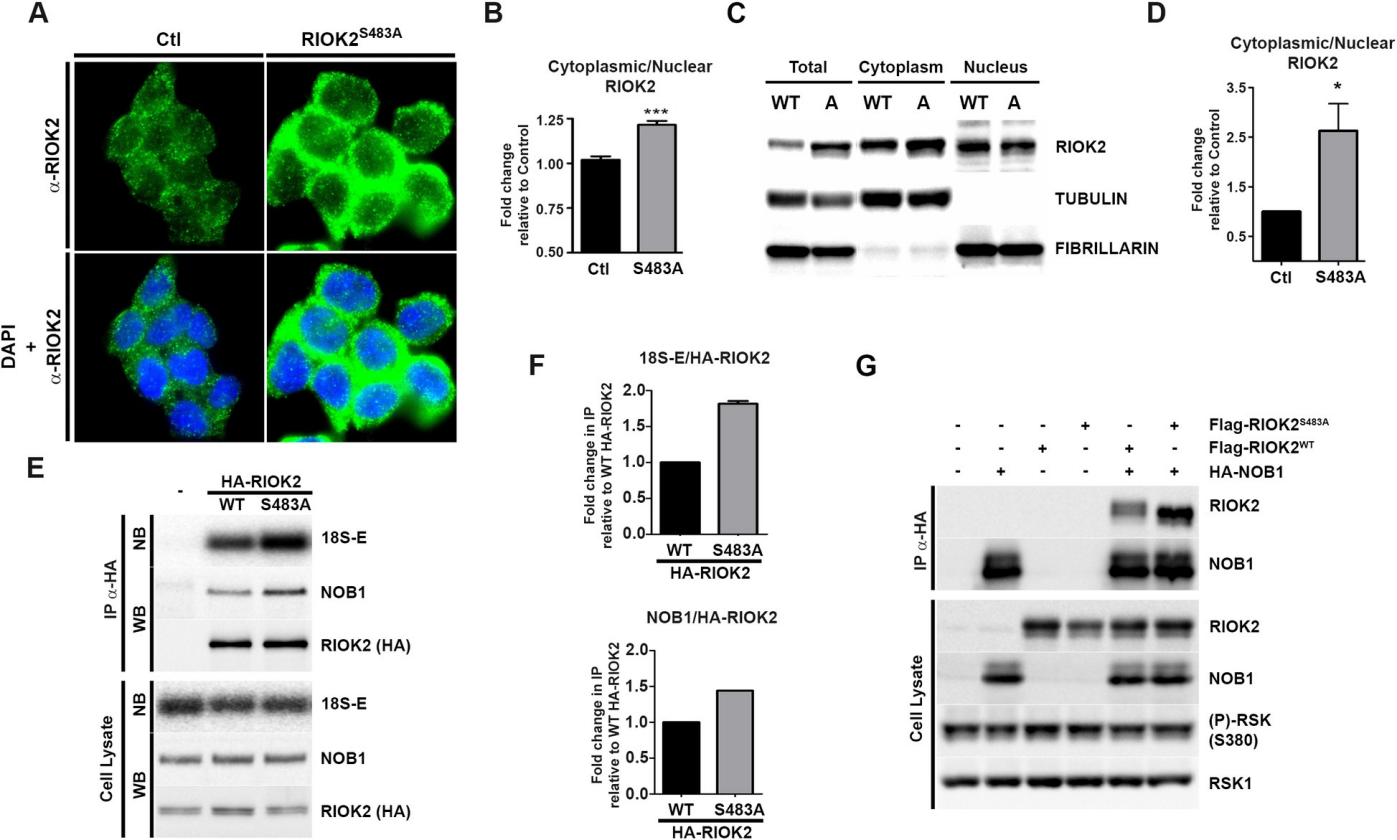
Loss of RIOK2 phosphorylation at Ser483 increases its association with cytoplasmic pre-40S particles. *(A)* RIOK2 localization was analyzed by IF microscopy using anti-RIOK2 antibodies in RIOK2^WT^ (Ctl) and RIOK2^S483A^ eHAP1 cell lines. Nuclei were visualized by DAPI staining. *(B)* Quantification of fluorescence observed in *(A)* using ImageJ software, as described in S7 Fig, and expressed as fold change relative to Ctl (n = 100 cells from different fields). Statistically significant differences are indicated by asterisks (***: P<0.0001, One-tailed Mann Whitney test). *(C)* Nucleo-cytoplasmic fractionation of serum-growing RIOK2^WT^ (Ctl) and RIOK2^S483A^ eHAP1 cell lines. RIOK2 levels in the different fractions (total cell extract, cytoplasm, nucleus) were analyzed by WB. Fractionation quality was validated using antibodies detecting tubulin (cytoplasmic protein) and fibrillarin (nuclear protein). *(D)* WB signals obtained in the cytoplasmic and nuclear fractions from *(C)* were quantified. The graph represents cytoplasmic/nuclear intensity ratios. Statistically significant differences are indicated by asterisks (*: P≤0.05, One-tailed Mann Whitney test). *(E)* HEK293 cells were transfected with plasmids expressing HA-tagged RIOK2^WT^ or RIOK2^S483A^, or with empty vector (-). HA-RIOK2 was immunoprecipitated and co-immunoprecipitated proteins and 18S-E pre-rRNA were analyzed by WB and NB, respectively. *(F)* Quantification of the WB and NB signals obtained in *(E)* expressed as fold change compared to immunoprecipitated HA-RIOK2^WT^. *(G)* HEK293 cells were co-transfected with plasmids expressing HA-NOB1 and either Flag-RIOK2^WT^ or Flag-RIOK2^S483A^. HA-NOB1 was immunoprecipitated and the co-immunoprecipitated proteins were analyzed by WB.

To elucidate the molecular impact of RSK-dependent RIOK2 phosphorylation during the maturation of pre-40S particles, we first compared the nucleo-cytoplasmic distribution of RIOK2^WT^ and RIOK2^S483A^ in eHAP1 cells using immunofluorescence (IF) microscopy (Fig 5A). Our results indicated that RIOK2^S483A^ accumulates in the cytoplasm to a greater extent than RIOK2^WT^. Quantification of the nuclear and cytoplasmic signals revealed a significant increase in the cytoplasmic to nuclear localization ratio of RIOK2^S483A^ compared to RIOK2^WT^ (Fig 5B). This observation was independently confirmed using cell fractionation assays. Protein samples were prepared from either total cell extracts or from the isolated cytoplasmic and nuclear fractions of eHAP1 cells expressing RIOK2^WT^ and RIOK2^S483A^. Western blotting analyses revealed that RIOK2^S483A^ was found more abundant in the cytoplasmic fraction than RIOK2^WT^ (Fig 5C and quantifications in Fig 5D). Altogether, these results indicate that in RIOK2^S483A^-expressing cells, both the mutant RIOK2 protein and pre-40S particles (Fig 4E and 4F) accumulate in the cytoplasm.

To more directly assess the physical association between RIOK2 and pre-40S particles, we performed immunoprecipitation (IP) experiments. We purified pre-40S particles from HEK293 cells expressing HA-tagged versions of RIOK2^S483A^ or RIOK2^WT^, and quantified the levels of co-purified 18S-E pre-rRNA by Northern blotting. Using this approach, we found that the 18S-E pre-rRNA co-immunoprecipitated with ~2-fold increased efficiency with RIOK2^S483A^ compared to RIOK2^WT^ (Fig 5E and quantifications in Fig 5F). Importantly, ectopic expression of RIOK2^S483A^ in these experiments did not change the global level of 18S-E pre-rRNA (Fig 5E, Cell Lysate), most likely due to the presence of endogenous RIOK2, strengthening the conclusion that 18S-E pre-rRNA is more efficiently co-purified with RIOK2^S483A^. We also monitored by western blot the presence in the precipitated particles of endogenous NOB1, another component of cytoplasmic pre-40S particles whose release occurs after RIOK2 dissociation. We found that NOB1 also co-immunoprecipitated with increased efficiency with RIOK2^S483A^ (Fig 5E and quantifications in Fig 5F). These results were confirmed by immunoprecipitating HA-tagged NOB1 from HEK293 cells also expressing Flag-tagged versions of either RIOK2^WT^ or RIOK2^S483A^ (Fig 5G). We observed that Flag-RIOK2^S483A^ was more efficiently co-purified with HA-NOB1 compared to Flag-RIOK2^WT^. Interestingly, we noticed in this experiment a shift in the electrophoretic mobility of Flag-RIOK2^WT^ that is lost with Flag-RIOK2^S483A^ mutant, supporting that RIOK2 is indeed post-translationally modified at Ser483. We conclude that the loss of RSK-mediated phosphorylation at Ser483 increases the steady-state association of RIOK2 with pre-40S particles.

### RIOK2 phosphorylation at Ser483 facilitates its release from pre-40S particles and re-import into the nucleus

**Fig 6:**
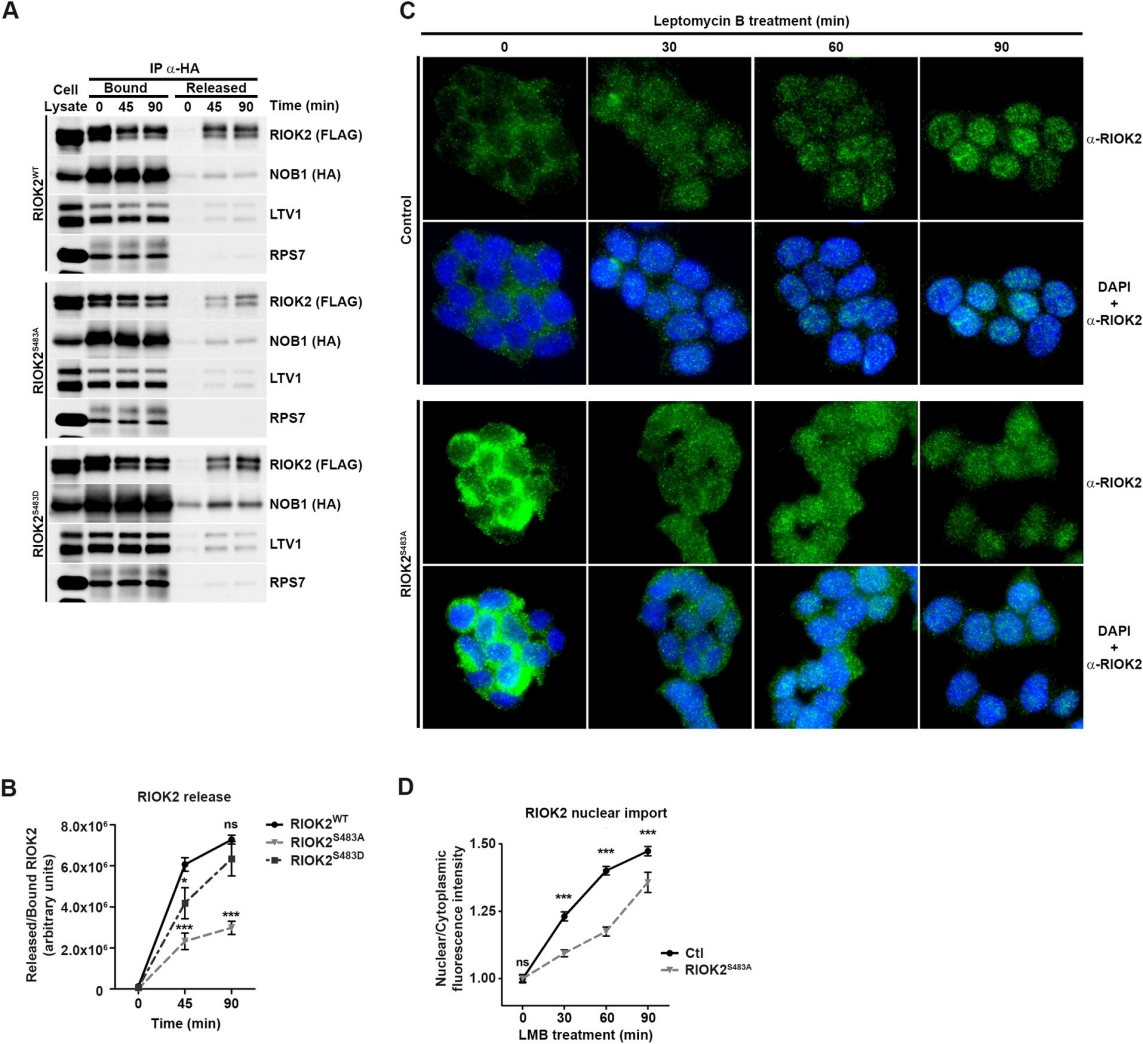
RIOK2 phosphorylation at Ser483 facilitates its release from pre-40S particles and re-import into the nucleus. *(A)* HEK293 cells were co-transfected with plasmids expressing HA-NOB1 and Flag-RIOK2^WT^, Flag-RIOK2^S483A^ or Flag-RIOK2^S483D^. Pre-40S particles were immunopurified via HA-NOB1 and subsequently incubated for 45 or 90 min with a buffer inducing RIOK2 release at 16°C. The presence of RIOK2, LTV1 and RPS7 proteins in supernatants (released proteins) and on beads (pre-40S-bound proteins) were analyzed by WB. Experiments with Flag-RIOK2^S483A^ and Flag-RIOK2^S483D^ were performed with different sets of Flag-RIOK2^WT^ as controls. A representative WB experiment for Flag-RIOK2^WT^ is shown. *(B)* Quantification of WB signals from *(A)* using ImageLab software and expressed as released over bound RIOK2 ratios. Statistically significant differences are indicated by asterisks (***: P≤0.001, **: P≤0.01, Two-way ANOVA test, Bonferroni posttests). *(C)* RIOK2^WT^ and RIOK2^S483A^ eHAP1 cells were treated with Leptomycin B (LMB, 20 nM) for the indicated times. Subcellular localization of RIOK2 was monitored by immunofluorescence microscopy using specific antibodies. Nuclei were visualized by DAPI staining. *(D)*, Quantification of nuclear to cytoplasmic fluorescence ratios at the indicated time points obtained in *(C)* using ImageJ software (n = 100 cells from different fields), as described in S7 Fig. Statistically significant differences are indicated by asterisks (***: P≤0.001, *: P≤0.05, 2way ANOVA tests, Bonferroni posttests).

To understand the molecular determinants accounting for the higher steady-state association of RIOK2^S483A^ with pre-40S particles, we performed *in vitro* RIOK2 dissociation assays from purified pre-40S particles (Fig 6A and quantifications in Fig 6B). For this, we purified pre-40S particles using HA-NOB1 as a bait from HEK293 cells also expressing Flag-tagged versions of either RIOK2^WT^, RIOK2^S483A^ or RIOK2^S483D^. The presence of the pre-40S particle AMF LTV1 and ribosomal protein RPS7 in the purified material besides HA-NOB1 and Flag-RIOK2 (Fig 6A) confirmed efficient purification of pre-40S particles. These pre-40S particles bound to the anti-HA affinity matrix were then incubated in a buffer intended to promote RIOK2 catalytic activity, which is required for its dissociation from pre-40S particles [57,67]. Using this approach, we found that the dissociation kinetics of RIOK2^S483A^ was significantly less efficient than RIOK2^WT^, as only ~30% of the mutant protein was dissociated from pre-40S particles compared to RIOK2^WT^ following a 90 min incubation (Fig 6B). In contrast, the phosphomimetic RIOK2^S483D^ mutant was found to display dissociation kinetics quite similar to RIOK2^WT^, since by 90 min of incubation, the same amounts of RIOK2^S483D^ and RIOK2^WT^ were released from pre-40S particles. Importantly, we noticed that the majority of LTV1 and RPS7 remained associated to NOB1-immunoprecipitated pre-40S particles, indicating that dissociation of RIOK2 is not due to artifactual release of pre-40S particles during incubation time. We concluded from these experiments that RIOK2^S483A^ is more stably associated to pre-40S particles compared to RIOK2^WT^ or RIOK2^S483D^, which suggests that phosphorylation of RIOK2 at Ser483 facilitates its release from cytoplasmic pre-40S particles.

We anticipated that defects in RIOK2 dissociation from cytoplasmic pre-40S particles would slow down its recycling to the nucleus. To investigate the dynamics of RIOK2 shuttling, we compared the kinetics of RIOK2^WT^ or RIOK2^S483A^ nuclear import upon inhibition of pre-40S particle export using leptomycin B (LMB), an inhibitor of CRM1 exportin. We monitored the progressive transfer of fluorescence from the cytoplasm to the nucleus following LMB treatment as a readout of the rate of dissociation of RIOK2 from cytoplasmic pre-40S particles and re-import in the nucleus. RIOK2^WT^ or RIOK2^S483A^ eHAP1 cell lines were treated with LMB and the nucleo-cytoplasmic distribution of RIOK2 was analyzed by IF during a time-course of LMB treatment (Fig 6C and quantifications in Fig 6D). In cells expressing RIOK2^WT^, the vast majority of cytoplasmic RIOK2 was imported back to the nucleus by 60 min of LMB treatment and the cytoplasmic signal became barely detectable by 90 min (Fig 6C). In contrast, RIOK2^S483A^ remained mostly cytoplasmic after 60 minutes of LMB treatment and a stronger signal remained in the cytoplasm after 90 min compared to RIOK2^WT^. Quantification of the nucleo-cytoplasmic ratios of the IF signals confirmed the slower nuclear import rate of RIOK2^S483A^ compared to RIOK2^WT^ (Fig 6D). We obtained similar results for NOB1, whose nuclear import following LMB treatment occurred more slowly in cells expressing RIOK2^S483A^ compared to control cells (S4A Fig and quantifications in S4B Fig). We concluded that phosphorylation at Ser483 facilitates the release of RIOK2 and NOB1 from cytoplasmic pre-40S particles and allows their recycling into the nucleus.

## Discussion

The MAPK signaling pathway ensures coordinated expression of ribosome components and of the machinery involved in pre-ribosome assembly and maturation [22]. Upon activation, the MAPK pathway promotes both rDNA transcription by Pol I and Pol III in the nucleus, and translation of mRNAs encoding RPs and AMFs in the cytoplasm. Our study provides evidence that MAPK signaling applies another level of coordination during ribosome biogenesis, by directly regulating pre-40S particle assembly and maturation. In addition, since ERK and RSK inhibition induce processing defects at different stages of the maturation process (for example a clear delay in the maturation of the 30S precursor, Fig 1), both kinases may also regulate several other steps of pre-rRNA processing.

**Fig 7:**
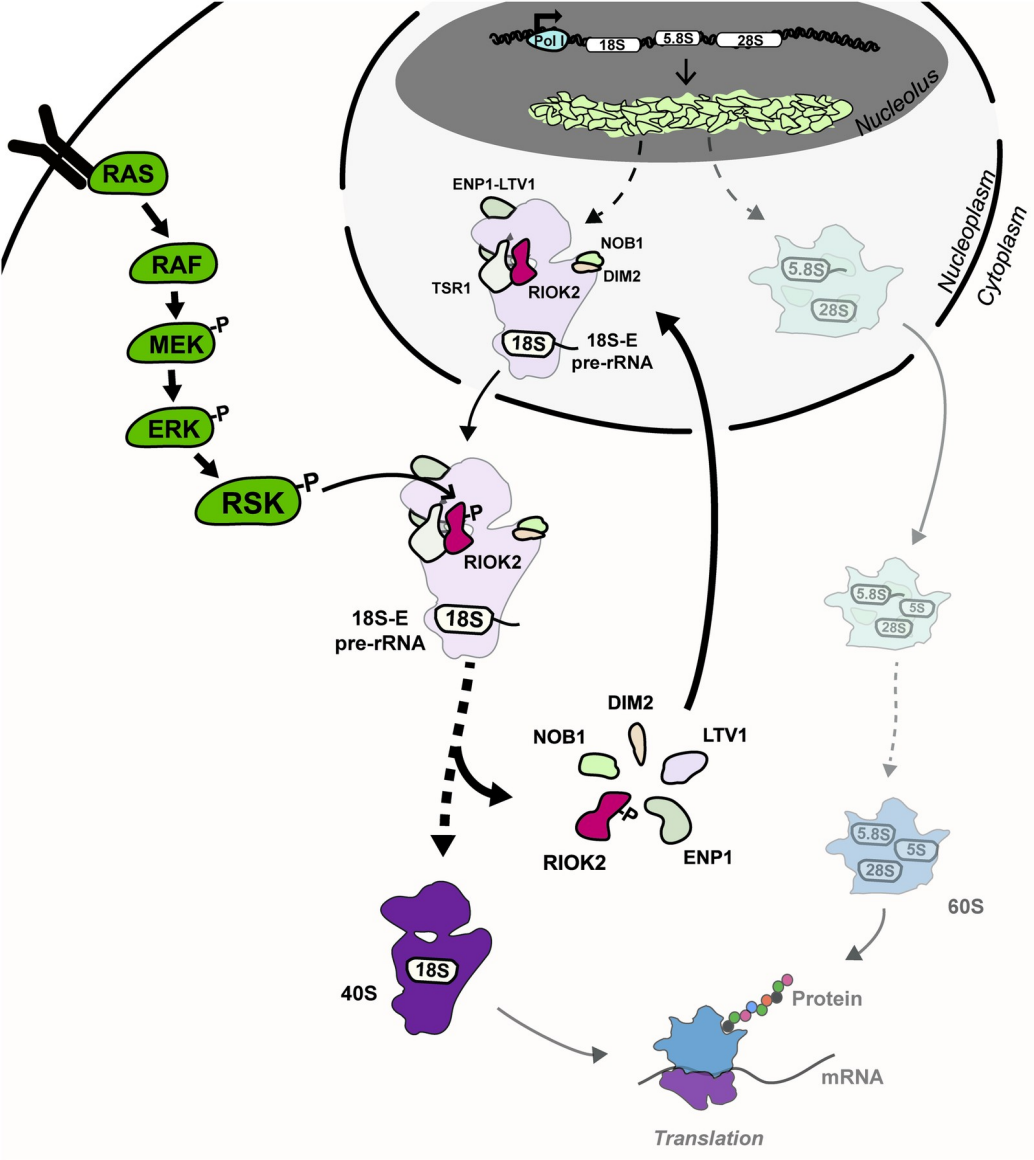
RIOK2 phosphorylation at Ser483 by RSK facilitates late stages of pre-40S particle maturation. RIOK2 is incorporated into pre-40S particles in the nucleus and participates to their export to the cytoplasm. Phosphorylation of RIOK2 by RSK at Ser483 facilitates its dissociation from pre-40S particles, which allows simultaneous or subsequent dissociation of other factors (ENP1, LTV1, DIM2, NOB1) to promote efficient maturation of the 18S-E pre-rRNA. This phosphorylation event is required for optimal protein synthesis and cell proliferation.

We report direct evidence showing that RSK stimulates the maturation of pre-40S particles, most likely by improving the dissociation efficiency of RIOK2 and of the other AMFs whose release depends on RIOK2 (such as NOB1 as shown in our study). These events are expected to facilitate the subsequent maturation steps towards completion of small subunit biogenesis (Fig 7). We identified RIOK2 as a direct phosphorylation target of RSK and used a combination of approaches to gain in-depth functional understanding of how phosphorylation by RSK influences RIOK2 function.

We demonstrated that RIOK2 phosphorylation at Ser483 stimulates the maturation of the 18S-E pre-rRNA by facilitating RIOK2 release from pre-40S particles and promoting its recycling to the nucleus. It remains unclear whether RSK phosphorylates RIOK2 within pre-40S particles or before its association. A recent cryo-electron microscopy (cryo-EM) structure of a late human pre-40S particle containing RIOK2 has been reported [68]. The domain of RIOK2 containing Ser483 is not resolved in the structure (from V300 to C493), suggesting that this domain is highly flexible. Given that the flanking residues (E299 and S494) are located on the external surface of the pre-40S particle, it seems that this domain is protruding outside of the protein, and would therefore be accessible to RSK kinase for phosphorylation (S5 Fig). These observations suggest that RSK could phosphorylate RIOK2 once incorporated into pre-40S particles to stimulate its dissociation. RSK has not been detected in pre-40S particles purified using different baits [69], suggesting that its interaction with pre-40S particles may be very transient or labile. Consistent with this, sucrose gradient experiments showed that RSK can be detected to low levels in the fractions containing pre-40S particles (S6 Fig).

The molecular mechanisms underlying yeast Rio2 release from pre-40S particles have been recently investigated [67,70]. Rio2 binds to pre-40S particles in a catalytically inactive conformation with its catalytic P-loop lysine (Lys106) bound to the pre-rRNA [59]. Following conformational rearrangements within the pre-40S particles involving the Rps20 connecting loop, release of the P-loop Lys106 allows Rio2 activation and dissociation from the particles. In the human pre-40S particle, RIOK2 seems to be positioned in a similar way compared to the yeast particle, with its P-loop Lys105 in close contact to helix 30 of the 18S rRNA (S5 Fig). However, as the C-terminal extension of RIOK2 containing Ser483 is not present in yeast Rio2, some aspects of the function or regulation of the protein are likely different in yeast and human cells. We propose that phosphorylation of RIOK2 at Ser483 by RSK could participate in conformational rearrangements that trigger RIOK2 catalytic activity by releasing its P-loop lysine and/or weakening its association with pre-40S particles, both favoring RIOK2 dissociation from pre-40S particles. Interestingly, although in yeast Ltv1 has been proposed to dissociate from pre-40S particles concomitantly to Rio2 through the connecting Rps20 loop [70], RIOK2 dissociated independently from LTV1 in our *in vitro* assay (Fig 6A). This result suggests that additional factors, not present in the pre-40S particles purified in our assay, may coordinate the dissociation of LTV1 and RIOK2. RIOK1 could be a candidate since it has been proposed that RIOK1 position on pre-40S particles overlaps the binding sites of both LTV1 and RIOK2 [68]. RIOK1 could therefore trigger structural rearrangements within pre-40S particle inducing LTV1 and RIOK2 release.

Part of the data supporting our conclusion that RIOK2 phosphorylation at Ser483 is required for optimal maturation of pre-40S particles stems from *in vivo* experiments where RIOK2 Ser483 was substituted to an alanine using the CRISPR/Cas9 approach. Although these mutant cells display phenotypes consistent with a partial loss of RIOK2 function, we cannot exclude that these effects are not due to the loss of phosphorylation, therefore that what is important for efficient 18S-E pre-rRNA processing is Ser483 *per se*, and not its phosphorylation status. However, we consider this hypothesis unlikely for several reasons. Substitution of Ser483 to an aspartic acid instead of an alanine (RIOK2^S483D^ mutant) does not induce the same processing defects. In particular, accumulation of 18S-E pre-rRNA is not observed (Fig 4C and 4D), indicating that the serine residue can be substituted by a residue mimicking the phosphorylated state without significant consequences on pre-40S particle maturation. Interestingly, the RIOK2^S483D^ mutant displays a slight accumulation of the 30S intermediate, suggesting that earlier stages of the pre-40S pathway are impaired by this mutation, possibly at the stage of RIOK2 recruitment into pre-ribosomes in the nucleus. Furthermore, in our i*n vitro* dissociation assay, RIOK2^S483D^ behaved like the wild-type protein, indicating again that a negative charge at position 483 is sufficient to confer close to wild-type physical properties to RIOK2. Finally, release of assembly factors by phosphorylation events is not unprecedented. In yeast, Hrr25 kinase phosphorylates Rps3, Enp1 and Ltv1 within nuclear pre-40S particles and thereby weakens their association with the particle to promote conformational rearrangements necessary for formation of the beak structure [70–72]. Hrr25/CK1 also promotes the dissociation of Ltv1 from pre-40S particles both in human and yeast [73,74]. These data combined with our study suggest that phosphorylation events are a common theme in the regulation of AMF dissociation from pre-ribosomal particles.

Our results show that mutation of RSK target site within RIOK2 (RIOK2^S483A^) induces processing defects that differ from those observed upon MAPK inhibition. RIOK2 is specifically involved in the late, cytoplasmic stages of the maturation of the pre-40S particles containing the 18S-E pre-rRNA, and alterations of RIOK2 function are therefore expected to impair the maturation of this precursor. In contrast, the MAPK pathway regulates ribosome biogenesis at multiple levels (S1A Fig). ERK and/or RSK inactivation is therefore expected to induce pleiotropic defects in ribosome biogenesis. Our data show that ERK inhibition (and consequently RSK inhibition) leads to the accumulation of the 30S intermediate, a phenotype similar to those obtained following depletion of early-associating ribosomal proteins, in particular RPS6 [75]. Since RPS6 is a known target of RSK kinase [45], we hypothesize that phosphorylation of RPS6 by RSK may be important for the incorporation of RPS6 into pre-ribosomes and that this may constitute another important regulation of pre-ribosome assembly by the MAPK pathway. This assumption is supported by recent findings in yeast, where inhibition of RPS6 phosphorylation was shown to prevent its incorporation into pre-ribosomes [76]. The accumulation of the 30S intermediate upon inactivation of ERK and RSK affects production of the downstream intermediates and thus precludes accumulation of 18S-E even if the activity of RIOK2 is affected. It is therefore biologically relevant to observe that inactivation of the MAPK pathway or RIOK2 does not result in the same processing defects, because the former has a broader impact on the maturation pathway than the latter.

Inhibition of RSK-dependent RIOK2 phosphorylation, although it delays 18S-E pre-rRNA processing, does not affect the steady-state amount of the small ribosomal subunit (18S rRNA levels). This possibly means that point mutation of S483 only partially affects RIOK2 function and delays the maturation of pre-40S particles to an extent that is not sufficient to impact mature 18S rRNA levels, as previously observed in other studies [77,78]. However, we do observe a global defect in translation correlated with a decrease in cell proliferation. Several hypotheses can be proposed to account for this apparent paradox. For technical reasons, we may have been unable to detect minor reductions in mature 18S rRNA levels. Another hypothesis could be that compensation mechanisms may operate to maintain mature 18S rRNA levels despite RIOK2 mutation. For example, mutated cells may extend the half-life of the ribosomes produced in these conditions to counterbalance a production defect. These ribosomes may become partially dysfunctional due to increased exposure to reactive oxygen species, leading to stalled ribosomes or decreased translation efficiency [79].

RIOK2 is the first pre-ribosome AMF shown to be regulated by the MAPK pathway. As RSK kinases share several phosphorylation targets with S6K and AKT, which belong to the PI3K/AKT/mTOR signaling pathway, it would be interesting to assess also the contribution of this pathway in the regulation of AMF functions. Since ribosome synthesis involves hundreds of AMFs, potentially many other MAPK- or PI3K/AKT/mTOR-driven regulatory mechanisms operate. Like RIOK2, a significant proportion of other AMFs corresponds to energy-consuming enzymes such as kinases, ATPases, GTPases or RNA helicases [1,3,4]. These enzymes have been suggested to provide directionality, accuracy and quality control to the process. They are believed to provide energy to overcome thermodynamically unfavorable steps of the process such as disruption of stable RNA helices, protein/RNA or protein/protein interactions [80,81]. Our *in silico* screen identified several other potential substrates of RSK and among these, energy-consuming enzymes are particularly represented. We therefore propose that MAPK signaling may participate in the coordination of the series of events occurring during pre-ribosome assembly and maturation by stimulating the activity of selected energy-consuming enzymes, thereby allowing to overcome rate-limiting steps. Interestingly, mammalian RIOK1 and RIOK3, also involved in pre-40S particle maturation [82,83] are not phosphorylated within a RSK consensus motif suggesting a specific regulation of RIOK2 by RSK.

Our study paves the way for future exploration of the regulation of ribosome biogenesis at the post-transcriptional level by the MAPK pathway. Identification of key limiting steps, in particular those catalyzed by discrete catalytic activities, would help in designing innovative molecules aimed at counteracting MAPK-driven deregulated ribosome production in pathologies, such as cancers or RASopathies.

## Materials and methods

### Cloning

Plasmids and sequences of oligonucleotides used in this study are listed in S2 Table. All clonings have been performed using In-Fusion HD Cloning Plus (Takara, Cat#638911) according to manufacturer’s recommendations. Transformations have been performed using Stellar™ Competent Cells (Takara, Cat#636763).

### Human cell lines, transfections and chemicals

HEK293 cells and HeLa cells were obtained from the American Type Culture Collection without further authentication. eHAP1 human cell line was purchased from Horizon Discovery. Human cells were maintained in 5% CO_2_ at 37°C. HEK293 and HeLa cells were cultured in Dulbecco’s modified Eagle’s medium (DMEM) and eHAP1 cells in Iscove Modified Dulbecco Media (IMDM). Both media were supplemented with 10% Fetal Bovine Serum, 1% Penicillin-Streptomycin, 1% Pyruvate. When indicated, cells were treated with Phorbol 12-Myristate 13-Acétate (Fisher Scientific, Cat#10061403), Human EGF (Euromedex, Cat# HC88823), LJH685 (Selleck Chemicals, S7870), BI-D1870 (Selleck Chemicals, S2843) and/or PD184352 (Selleck Chemicals, S1020). For transient plasmid expression, cells were transfected using either Jet Prime reagent or calcium phosphate precipitation. For shRNA-mediated RSK1/2 knockdown, cells were infected by lentiviruses produced with vectors from the Mission TRC shRNA library (RSK1, TRCN470; RSK2, TRCN537) in the presence of 4 mg/ml polybrene and selected 48 h after infection with 2 μg/mL puromycin.

### Antibodies

Anti-phospho Ser483 RIOK2 antibodies were generated according to immunization protocol from Covalab. Briefly, a peptide phosphorylated at Ser483 (MNQYRTRTL(Sp)ITS) conjugated to a carrier protein was first used to immunize rabbits. Following the final bleed, the immune serum was loaded onto a column with the control peptide (MNQYRTRTLSITS) coupled to agarose beads, thus retaining unmodified peptide-specific antibodies. The flow-through was then loaded onto a column with the modified peptide coupled to agarose beads, thus retaining modified peptide-specific antibodies. After elution, the eluate was assayed by ELISA against both peptides to control its immuno-reactivity and its specificity against the modification. Anti-phospho-ERK 1/2 (T202/Y204, #4370), anti-phospho-RSK (S380), anti-phospho-(Ser/Thr) Akt Substrate (#9614) and anti-ERK1/2 (#4695) antibodies were purchased from Cell Signaling Technology. Anti-RSK1(#GTX111050), anti-RSK2 (#38–6800), anti-puromycin (clone 12D10, #MABE343) and anti-HA (clone 16B12, #MMS-101R-500) antibodies were purchased from Genetex, Life Technologies, Millipore and Covance, respectively. Anti-RIOK2 (#A302-450A) and anti-ENP1 (#A304-568A) antibodies were purchased from Bethyl Laboratories.

### CRISPR/Cas9 genome editing

RIOK2^S483A^ and RIOK2^S483D^ eHAP1 mutant cell lines were generated using CRISPR/Cas9-mediated double strand break and homologous recombination, using ouabain co-selection as described [62]. Oligos designed to encode the Cas9 guide RNA (http://crispor.tefor.net/) were annealed and ligated into BbsI-digested Addgene #86613 plasmid, resulting in plasmid 86613-RIOK2-gRNA. Single-stranded donor templates were designed to introduce the RIOK2^S483A^ or RIOK2^S483D^ point mutations along with silent mutations introducing an MscI or EcoRV restriction site, respectively. Plasmid 86613-RIOK2-gRNA and single-stranded donor templates for introduction of ouabain resistance and either RIOK2^S483A^ or RIOK2^S483D^ point mutations were electroporated into eHAP1 cells at 300 V with a Gene Pulser System (Bio-Rad Laboratories) in cuvettes with a 4-mm inter-electrode distance (Eurogentec). Transfected cells were grown for 48 h in the presence of 7 μM ouabain (Sigma, O3125; CAS:11018-89-6), then diluted into 14 cm-dishes and grown for 2–3 weeks in the presence of ouabain to obtain isolated clones. Clonal populations were isolated into 12 well-plates. To identify mutant cells, genomic DNAs were isolated from clonal populations using GenElute™ Mammalian Genomic DNA Miniprep Kit Protocol (Sigma-Aldrich). A genomic region of RIOK2 gene encompassing Ser483 was PCR-amplified from the genomic DNAs, and the presence of point mutations was revealed by digestion of PCR products with MscI (for RIOK2^S483A^) or EcoRV (for RIOK2^S483D^). Homozygous knock in clones were then confirmed by sequencing (Eurofins Genomics). The wild-type controls used in the study are randomly chosen eHAP1 cell lines electroporated with 86613-RIOK2-gRNA plasmid and donor templates but in which RIOK2 locus had not been edited.

### Proliferation and cell death analyses

Cell proliferation was assessed using either MTS assay (CellTiter 96 AQueous One Solution Cell Proliferation Assay, Promega, data are expressed as means of three repeated measures using at least 5 distinct samples for each condition) or cell counting (data are expressed as means of three repeated measures using 3 distinct samples for each condition). Cell death was monitored using FITC Annexin V Apoptosis Detection Kit (BioLegend). Populations of cells in apoptosis, necrosis or post-apoptosis were discriminated using a FACS Verse analyzer (BD Biosciences, data are expressed as means of 3 distinct samples for each condition).

### Protein synthesis assay

Global protein synthesis was determined using Sunset method [63]. Puromycin (InVivoGen, Cat#ant-pr; CAS: 58-58-2) was added in the culture medium (1μM) and cells were incubated for 20, 30 or 40 min at 37°C. After cell lysis, normalized amounts of total proteins were analyzed by Western Blot using anti-puromycin antibodies.

### RNA analyses

Extractions of total RNAs were performed using TRI REAGENT (MRC). After addition of 0.3 mL of chlorophorm per mL of TRI REAGENT, the mixtures were shaken vigorously and centrifuged at 12,000 g for 15 minutes at 4°C. Aqueous phases were submitted to a second round of extraction using 0.5 volume or water-saturated phenol and 0.5 volume of chloroform. For precipitation, RNAs were mixed with one volume of 2-propanol and incubated 10 min at room temperature. RNAs were pelleted by centrifugation at 12,000 × g for 10 minutes at 2–8°C. RNA pellets were washed with 1 mL of 75% ethanol and centrifugation at 12,000 × g for 10 minutes at 2–8°C. Air-dried pellets were resuspended with ultrapure MilliQ H_2_O and RNAs were quantified using a NanoDrop Spectrophotometer (Thermo Fisher Scientific). Northern blot experiments were performed as described in “Molecular Cloning”, Sambrook and Russell, CSHL Press (“Separation of RNA According to Size: Electrophoresis of Glyoxylated RNA through Agarose Gels”). Briefly, equal amounts of total RNAs (usually 4 μg for analysis of rRNA precursors or 1 μg for mature rRNAs) were mixed with five volumes of Glyoxal loading buffer [prepared by mixing the following: 6 ml DMSO, 2 ml deionized glyoxal, 1.2 ml 10X BPTE (see below), 600 μl 80% glycerol, 40 μl 10 mg/ml Ethidium Bromide]. The samples were heated 1 hour at 55°C and RNAs were separated by electrophoresis on 1.2% agarose gels in 1X BPTE running buffer [100 mM PIPES, 300 mM BIS-TRIS, 10 mM EDTA]. After electrophoresis, the gels were (i) rinsed 2 times 5 min with ultrapure MilliQ H_2_O, (ii) soaked 20 min at room temperature (RT) in 75 mM NaOH with gentle shaking to partially hydrolyze RNAs, (iii) rinsed 2 times 5 min with ultrapure MilliQ H_2_O, (iv) soaked 2 times 15 min at RT in [0.5 M Tris-HCl pH 7.4, 1.5 M NaCl] with gentle shaking to neutralize the pH, (v) Soaked 2 times 10 min at RT in 10X SSC with gentle shaking. RNAs were then transferred over night to Amersham Hybond N^+^ membranes (GE Healthcare) by capillarity with 10X SSC transfer buffer. Membranes were then exposed to 0.125 joules of 365 nm UV rays to crosslink RNAs on the membranes. Membranes were then hybridized with ^32^P-labeled oligonucleotide probes using the Rapid-hyb buffer (GE Healthcare). Radioactive membranes were exposed to PhosphorImager screens and signals were revealed using Typhoon imager (GE Healthcare). The sequences of the probes used to detect (pre-)rRNAs are described in S2 Table. Quantification of RNA levels was obtained using MultiGauge software (Fujifilm). Analyses of pre-rRNA precursor levels were performed using Ratio Analysis of Multiple Precursors (RAMP), as previously described [46].

### Metabolic labeling experiments

HEK293 cells were grown in 6-well plates at about 80% confluence and starved by overnight serum deprivation. Cells were then incubated in serum- and phosphate-free DMEM medium (Gibco, 11971–025) for 1 hour at 37°C and labelled for 1 hour at 37°C with 15 μCi of ^32^P-labelled orthophosphate (RADIOA BIOACTIF, P-RB-1). Cells were rinsed twice with 1 ml of 37°C-prewarmed, serum-free, phosphate-containing DMEM and incubated for 0 (immediately rinsed with ice-cold PBS), 60 or 180 min in serum-free DMEM (containing cold phosphate). When indicated, cells were treated with PD184352 inhibitor (20 μM) during the incubation in phosphate-free medium and during the labelling time (2 hours before time 0). PMA stimulation was started during the labelling time (1 hour before time 0). After the chase times, cells were rinsed twice with ice-cold PBS and lysed with 1 ml TRI-Reagent (MRC). Total RNAs were extracted using TRI-Reagent as described in “RNA analyses” and their concentration were determined using a Qubit fluorometer (Thermo Fisher Scientific). RNA samples were separated on a 1.2% agarose gel and transferred to a nylon membrane as described in “RNA analyses”. The membranes were exposed to PhosphorImager screens and signals were quantified using MultiGauge software.

### Protein analyses

Protein extracts were prepared as follows: cells were washed with ice-cold PBS, and lysed with Buffer A [10 mM K_3_PO_4_, 1 mM EDTA, 5 mM EGTA, 10 mM MgCl_2_, 50 mM β-glycerophosphate, 0.5% Nonidet P-40, 0.1% Brij 35, cOmplete protease inhibitor cocktail (Roche), Phosphatase Inhibitor Cocktail 2 and 3 (Sigma-Aldrich)]. Protein concentrations were measured using Bio-Rad Protein Assay and normalized protein concentrations were resuspended in Laemmli Buffer [40 mM Trizma base, 2% SDS, 5% Glycerol, 0.08% Bromophenol blue, 25 mM DTT]. Western blot experiments were performed as follow: protein samples were heated 5 min at 95°C, loaded on SDS-polyacrylamide gels (8 to 10%) and transferred to nitrocellulose membranes using Trans-blot turbo transfer system (Bio-Rad). Membranes were saturated for 1 h with TBST buffer (150 mM NaCl, 20 mM Tris pH 8.0, 0.001% Tween-20) containing 5% powder milk, and incubated over night with the same buffer containing primary antibodies. After 3 washes with TBST buffer, membranes were incubated for 1 h with the secondary antibodies diluted in TBST containing 5% powder milk, and washed three times with TBST buffer before ECL detection. ImageLab software (Biorad) was used to quantify protein signals from Western Blot.

### In-gel tryptic digestion and nanoLC-MS/MS analysis

For mass spectrometry analysis, HA-RIOK2 immunoprecipitated samples, prepared in triple biological replicates for each condition, were reduced for 30 min at 55°C in Laemmli buffer containing 25 mM DTT and alkylated in 90 mM iodoacetamide for 30 min in the dark at room temperature. Equal volumes of samples were separated by SDS–PAGE on 10% polyacrylamide gels, followed by gel staining with InstantBlue (Expedeon Protein Solutions) according to the manufacturer’s instructions. Bands at the molecular weight of HA-RIOK2 were excised and subjected to in-gel tryptic digestion using modified porcine trypsin (Promega) at 20 ng/μl as previously described (Shevchenko et al., 1996). The dried peptide extracts obtained were resuspended in 21 μl of 0.05% trifluoroacetic acid in 2% acetonitrile spiked-in with 0.1X final concentration of iRT standard peptides (Biognosis) and analyzed by online nanoLC using UltiMate 3000 RSLCnano LC system (ThermoScientific, Dionex) coupled to an Orbitrap Fusion Tribrid mass spectrometer (Thermo Scientific, Bremen, Germany). 5μl of each peptide extracts were loaded onto 300 μm ID x 5 mm PepMap C18 precolumn (ThermoFisher, Dionex) at 20 μl/min in 2% acetonitrile, 0.05% trifluoroacetic acid. After 5 min of desalting, peptides were online separated on a 75 μm ID x 50 cm C18 column (in-house packed with Reprosil C18-AQ Pur 3 μm resin, Dr. Maisch; Proxeon Biosystems, Odense, Denmark), equilibrated in 95% of buffer A (0.2% formic acid), with a gradient of 5 to 25% of buffer B (80% acetonitrile, 0.2% formic acid) for 80 min then 25% to 50% for 30 min at a flow rate of 300 nl/min. The instrument was operated in the data-dependent acquisition (DDA) mode using a top-speed approach (cycle time of 3 s). The survey scans MS were performed in the Orbitrap over m/z 350–1550 with a resolution of 120,000 (at 200 m/z), an automatic gain control (AGC) target value of 4e5, and a maximum injection time of 50 ms. Most intense ions per survey scan were selected at 1.6 m/z with the quadrupole and fragmented by Higher Energy Collisional Dissociation (HCD). The monoisotopic precursor selection was turned on, the intensity threshold for fragmentation was set to 50,000 and the normalized collision energy was set to 35%. The resulting fragments were analyzed in the Orbitrap with a resolution of 30,000 (at 200 m/z), an automatic gain control (AGC) target value of 5e4, and a maximum injection time of 60 ms. The dynamic exclusion duration was set to 30 s with a 10 ppm tolerance around the selected precursor and its isotopes. For internal calibration the 445.120025 ion was used as lock mass. Triplicate technical LC-MS measurements were performed for each sample.

### Database search and label-free quantitative analysis of RIOK2 phosphorylation

All raw MS files were processed with MaxQuant (v 1.5.2.8) for database search with the Andromeda search engine and quantitative analysis. Data were searched against the UniProtKB/Swiss-Prot protein database released 2015_07 with *Homo sapiens* taxonomy (11953 sequences) supplemented with the human 3HA-RIOK2 sequence, the Biognosys iRT peptide sequences and a list of frequently observed contaminant sequences provided in MaxQuant 1.5.2.8. Carbamidomethylation of cysteines was set as a fixed modification, whereas oxidation of methionine, protein N-terminal acetylation, and phosphorylation of serine, threonine, and tyrosine were set as variable modifications. Enzyme specificity was set to trypsin/P, and a maximum of three missed cleavages was allowed. The precursor mass tolerance was set to 20 ppm for the first search and 10 ppm for the main Andromeda database search, and the mass tolerance in MS/MS mode was set to 0.025 Da. The required minimum peptide length was seven amino acids, and the minimum number of unique peptides was set to one. Andromeda results were validated by the target-decoy approach using a reverse database and the false discovery rates at the peptide-spectrum matches (PSM), protein and site levels were set to 1%. Phosphosite localization was evaluated on the basis of the Phosphosite Localization Scoring and Localization Probability algorithm of the Andromeda search engine. For label-free relative quantification of the samples, the match between runs option of MaxQuant was enabled with a time window of 2 min, to allow cross-assignment of MS features detected in the different runs. Relative quantification of RIOK2 phosphorylation sites was performed by retrieving the intensity values of the phosphorylated peptide ions from the MaxQuant evidence.txt output that contains quantitative data for all peptide ions. Intensity values were first normalized for instrument variation using the MS intensities of the iRT spiked-in standards. The variability that may occur during the immunopurification was then corrected in each sample by normalizing the iRT-normalized intensity values to that of the HA-RIOK2 bait. This second normalization was performed based on the sum of the intensity values of HA-RIOK2 tryptic peptides and, to exclude variations resulting from RIOK2 phosphorylation, all of the HA-RIOK2 peptides containing a residue susceptible to phosphorylation were eliminated from the calculation. Mean intensity values were then calculated from technical LC-MS replicates. To evaluate the relative abundance of phosphorylation at a given site, total areas of tryptic peptides encompassing the site were calculated for the phosphorylated forms by aggregating data corresponding to peptide ions charge states (2+ and 3+), modification other than phosphorylation (oxidized methionine), and tryptic miscleavages (overlapping sequences).

### Immunoprecipitations

HEK293 cells (one 10 cm-dish at 90% confluence per condition) were lysed in buffer A supplemented with 0.1 M KCl. Cell lysates were incubated with the indicated antibodies for 1 h 45 min, then with protein A-Sepharose CL-4B beads (GE Healthcare) for another 45 min. Immunoprecipitates were washed 3 times with lysis buffer and eluted from the beads upon addition of Laemmli buffer and incubation 5 min at 95°C.

### Nucleo-cytoplasmic fractionation assay

Control (CTL) and RIOK2^S483A^ eHAP1 cells were lysed in Buffer B [20 mM Tris-HCl pH 7.5, 1.5 mM MgCl_2_, 10 mM KCl, 0,05% NP40, 1 mM DTT, 1X complete protease inhibitor cocktail (Roche)]. After saving a total extract sample, cell extracts were incubated on ice for 5 min and nuclei were pelleted by centrifugation at 200 g for 5 min at 4°C. The cytoplasmic fractions (supernatants) were collected. Pellets containing nuclei were first washed in a buffer solution containing 0.25 M sucrose, 3.3 mM MgCl_2_, and 10 mM Tris-HCl pH7.5. Pellets were then resuspended in sucrose solution 1 (250 mM sucrose, 10 mM MgCl_2_), deposited on a sucrose solution 2 (350 mM sucrose, 0.5 mM MgCl_2_) and centrifuged at 200 g for 5 min at 4°C. Pellets containing the nuclear fractions were resuspended in Buffer B. The total and nuclear fractions were sonicated (5 cycles of 30 sec ON/30 sec OFF, at 4°C with a Bioruptor Plus from Diagenode) and clarified by centrifugation at 13 000 rpm for 5 min at 4°C. Protein concentration of each fraction was measured using Bio-Rad Protein Assay and normalized protein concentrations were resuspended in Laemmli Buffer for immunoblotting experiments.

### In vitro dissociation assay

Pre-40S particles were immunoprecipitated as described above from HEK293 cells expressing Flag-RIOK2 and HA-NOB1 (one 10 cm-dish at 90% confluence per condition). After the third wash with buffer A supplemented with 0.1M KCl, beads were washed once with “RIOK2 dissociation buffer” (200 mM NaCl, 25 mM Tris-HCl pH 7,4, 10 mM MgCl_2_, 5 mM β-glycerophosphate). HA-NOB1-associated pre-40S particles were then incubated for indicated times in “RIOK2 dissociation buffer” supplemented with 1mM ATP at 16°C. Beads and supernatants were then separated and mixed with Laemmli buffer.

### Sucrose gradient fractionation

Three days after seeding, culture medium of two 15 cm-dishes of HEK293 cells at 90% confluence was removed and fresh 37°C-prewarmed medium was added to the cells. After an incubation of ∼90 min at 37°C, 10 μg/ml cycloheximide was added directly to the culture medium and incubation was prolonged for 10 min. Cells were harvested with trypsin and washed 2 times with ice-cold PBS supplemented with 10 μg/ml cycloheximide. The cell pellet was then washed with buffer B (10 mM HEPES–KOH pH 7.9, 10 mM KCl, 1.5 mM MgCl2, 100 μg/ml cycloheximide) and incubated 20 min on ice in buffer B supplemented with 0.5 mM dithiothreitol, 1 × cOmplete EDTA-free protease inhibitor cocktail (Roche) and 0.5 U/μl RNasin (Promega). After incubation cells were disrupted using a Dounce homogenizer with a tight pestle and centrifuged at 1000 x g for 10 min at 4°C, in order to pellet nuclei. The top soluble phase, containing the cytoplasmic fraction, was clarified through one centrifugation at 10 000 x g for 15 min at 4°C and quantified by measuring absorbance at 260 nm. Normalized amounts of extracts were loaded on a 10–50% sucrose gradient in buffer B. Gradients were centrifuged at 39 000 rpm for 2.5 h at 4°C in an Optima L-100XP ultracentrifuge (Beckman–Coulter) using the SW41Ti rotor with brake. Following centrifugation, the fractions were collected using a Foxy Jr fraction collector (Teledyne ISCO) and the absorbance at 254 nm was measured with a UA-6 device (Teledyne ISCO). For protein analyses, fractions were precipitated with TCA and protein pellets were resuspended in Laemmli buffer.

### Microscopy

For all microscopy experiments, cells were seeded on microscope cover glasses in 6-well plates and grown for 48 to 72 h. Immunofluorescence (IF) microscopy experiments were performed as described previously [57]. Briefly, after fixation in 4% paraformaldehyde (PFA), cells were permeabilized with [0.1% Triton X-100 and 0.02% SDS in PBS] for 5 min. Fixed cells were incubated in blocking solution [2% BSA (Sigma A8022) in PBS for 30 min and then incubated for 1h with the same solution containing primary antibodies diluted to 1:2000. Cells were washed 3 times for 5 min with [2% BSA in PBS], and subsequently incubated for 30 min with secondary antibodies (Alexa Fluor 488-conjugated goat anti-rabbit antibodies) diluted in blocking solution. After 3 washes, cells were incubated briefly in [0.1% Triton X-100, 0.02% SDS in PBS], and post-fixed with 4% PFA. After a wash with PBS, coverslips were mounted in VectaShield (Vector Laboratories). Fluorescent *In Situ* Hybridization (FISH) experiments were done as described previously [75]. Cells were fixed in 4% PFA, and after 2 washes with PBS, cells were permeabilized at 4°C for 18 h in 70% ethanol. Permeabilized cells were washed twice in (2X SSC, 10% formamide) and hybridized at 37°C in the dark for ≥ 5 h in hybridization buffer (10% formamide, 0.1X SSC, 0.5 mg/ml *E*. *coli* tRNAs, 10% dextran sulfate, 250 μg/ml BSA, 10 mM ribonucleoside vanadyl complexes, 0.5 ng/μL of Cy3-conjugated 5’ITS1 probe). After 2 washes in (2X SSC, 10% formamide), cells were rinsed with PBS, and coverslip were mounted in VectaShield. Images were captured using an inverted Olympus IX81 epifluorescence microscope equipped with a X100 objective lens (UPlan SApo 1.4 oil), a SpectraX illumination system (Lumencore) and a CMOS camera (Hamamatsu© ORCA-Flash 4.0), driven by MetaMorph (Molecular Devices). Fluorescent signals were captured after different exposure times (between 500 and 2000 ms) depending on signal intensities. Image analyses were performed using ImageJ software. The procedure used to quantify IF experiments is described in S7 Fig.

### In vitro kinase assay

For RSK kinase assays, human recombinant-activated RSK1 purchased from SignalChem (Catalog # R15-10G) was used with bacterially purified recombinant GST-RIOK2 (aa 443–552) as substrate (WT and S483A), under linear assay conditions. Assays were performed for 10 min at 30°C in kinase buffer [25 mmol/L Tris-HCl (pH 7.4), 10 mmol/L MgCl_2_, and 5 mmol/L β-glycerophosphate] supplemented with 5 μCi of [γ-^32^P]ATP. All samples were subjected to SDS-PAGE followed by immunoblotting, and incorporation of radioactive ^32^P label was determined by autoradiography using a Fuji PhosphorImager with ImageQuant software. The data presented are representative of at least three independent experiments.

### Statistical analyses

Data are expressed as means ± SEM. All statistical data (n≥3) were calculated using GraphPad Prism 5.01. Statistical details and significance reports can be found in the corresponding figure legends.

## Supporting information

S1 Fig *(A)* Schematic representation of pre-rRNA processing in human cells, adapted from [1] with precursor subcellular localization from [64]. Position of the probes (ITS1 and ITS2) used in Northern blot (NB) experiments to detect the pre-rRNAs are indicated. The known targets of ERK and RSK kinases involved in ribosome synthesis and translation are mentioned. The MAPK pathway promotes ribosome biogenesis through the regulation of multiple stages of the process, although no pre-ribosome assembly and maturation factor has been identified so far as a direct target of ERK or RSK kinases. *(B)* Schematic representation of the Ras/MAPK signaling pathway with pharmacological agonists and inhibitors used in this study. *(C)* RAMP analyses of 18S/28S ratios detected in Fig 1A after signal quantifications using MultiGauge software (Fujifilm).
(TIF)

S2 Fig *(A)* Schematic location of RIOK2 Ser483 with respect to the known functional domains of the protein. *(B)* Endogenous RIOK2 was immunoprecipitated from serum-growing HEK293 cells using anti-RIOK2 antibodies. A control immunoprecipitation was performed in parallel using IgGs (IgG) to validate the specificity of the immunoprecipitation. After immunoprecipitation, samples were treated (+) or not (-) with λ phosphatase and analyzed by WB using the indicated antibodies. *(C)* HA-RIOK2 was immunoprecipitated from serum-starved (Ctl) HEK293 cells treated (PD+PMA) or not (PMA) with PD184352 (10 μM, 1h) prior to PMA stimulation (100 ng/ml, 20 min). Purified HA-RIOK2 was isolated following SDS-PAGE and Coomassie staining, in gel digested with trypsin and the resulting peptides were submitted to nano LC-MS/MS analysis. *(D)* HCD MS/MS spectrum of the S483 monophosphorylated peptide (doubly charged precursor ion, MH3+, at m/z 1157.5670). Highlighted in red are site-determining ions and the corresponding peaks in the spectrum. In blue are indicated fragment ions that confirm the site localization and exclude another potential site. P: loss of H3PO4 from sequence ions. pS: phosphorylated serine residue. Cam: carbamidomethylated cysteine residue. *(E)* Partial amino acid sequences of RIOK2 proteins from the organisms indicated on the left were aligned using Jalview 2.11.0 software and Muscle with default parameters. The bottom consensus sequence was auto-calculated. The conserved RXRXXpS motif is boxed and Ser483 of human RIOK2 is indicated.
(TIF)

S3 Fig *(A)* Cell counting was performed on cultures of control (Ctl) and RIOK2^S483A^ (1 to 3) eHAP1 cell lines at the indicated time points with a cell counter (Beckman-Coulter Z1). *(B)* MTS assays were performed on control eHAP1 cell line cultured in the presence of PD184352 (10 μM), BI-D1870 (2,5 μM), or LJH685 (10 μM). ODs at 490 nm were measured using Spectramax at the indicated time points. For *(A)* and *(B)*, statistically significant differences between Ctl and other conditions are indicated by asterisks (***: P≤0.001, 2way ANOVA tests, Bonferroni posttests). *(C)* Apoptosis levels in cell lines from *(A)* were measured based on FITC-Annexin5 (FITC-A) and Propidium Iodide labelling. Apoptosis levels corresponding to FITC-A single-positive cells were monitored by FACS Verse analyzer and quantifications are expressed as percentage of total cells. No statistical difference between Ctl and RIOK2^S483A^ cell lines was revealed (One-tailed Mann Whitney test). *(D)* Control (Ctl) and RIOK2^S483A^ (1 to 3) eHAP1 cell lines were incubated with 1 μM puromycin for the indicated times. Protein extracts were analyzed by WB using anti-puromycin antibodies (left). Equal amounts of total proteins in each sample were controlled on TGX Stain-Free gels (Bio-Rad, right). *(E)* eHAP1 cells were transfected with siRNA targeting an irrelevant sequence (Ctl) or RIOK2. Total RNAs were extracted and pre-rRNA levels were monitored by NB. RIOK2 depletion was controlled by WB. *(F)* RAMP analyses of pre-rRNA levels detected in *(E)*, as in Fig 1. *(G)* Total cellular RNAs were extracted from control (Ctl), RIOK2^S483A^ or RIOK2^S483D^ eHAP1 cell lines. Accumulation levels of large subunit pre-rRNAs (ITS2 probe) and mature 28S rRNA were analyzed by NB. *(H)* RAMP analyses of 18S/28S ratios detected in *(G)* and Fig 4C, as in Fig 1. No statistical difference between Ctl RIOK2^S483A^ and RIOK2^S483D^ cell lines was revealed (Kruskal-Wallis test, Dunn’s multiple comparison test)
(TIF)

S4 Fig *(A)* RIOK2^WT^ and RIOK2^S483A^ eHAP1 cells were treated with Leptomycin B (LMB, 20 nM) for the indicated times. Subcellular localization of NOB1 was monitored by immunofluorescence microscopy using specific antibodies. Nuclei were visualized by DAPI staining. *(B)* Quantification of nuclear to cytoplasmic fluorescence ratios at the indicated time points obtained in *(A)* using ImageJ software (n = 100 cells from different fields), as described in Materials and Methods section and S7 Fig. Statistically significant differences are indicated by asterisks (***: P≤0.001, *: P≤0.05, 2way ANOVA tests, Bonferroni posttests).
(TIF)

S5 Fig Different views of the cryo-EM structure of RIOK2-bound pre-40S particles adapted from [68] (State C, PDB accession number 6G18). RIOK2 is coloured in purple, its catalytic domain highlighted in green and the P-loop Lys105 in orange. The protein domain comprised between Val300 to Cys493, containing Ser483, is not resolved in the structure. The flanking residues Glu299 and Ser494 are coloured in red. Helix 80 of the 18S rRNA (residues 1226–1237 and 1520–1532) has been coloured in yellow. *(A)* Global view of RIOK2-bound pre-40S particle. *(B)* Closer view of RIOK2 with the residues flanking the domain containing Ser483 (not resolved) at the surface of the particle. *(C)* Closer view of the catalytic domain showing that catalytic P-loop lysine 105 is in close contact to helix 30 of the 18S rRNA.
(TIF)

S6 Fig HEK293 cell extract was centrifuged through a 10 to 50% sucrose gradient. Proteins were TCA-precipitated from collected fractions and the proteins indicated on the right were detected by WB.
(TIF)

S7 Fig Quantification of fluorescence intensities following microscopy analyses (FISH and IF). RIOK2 nuclear and cytoplasmic signals have been quantified with ImageJ software as follows. *(A)* Nuclear masks (binary images) were generated from DAPI signal intensities and transposed to the corresponding α-RIOK2 immunofluorescence images to quantify the nuclear intensities. *(B)* To quantify α-RIOK2 cytoplasmic intensities, cytoplasmic masks were obtained by subtracting nuclear masks intensities to whole cell α-RIOK2 signals. *(C)* All signals were expressed as mean of fluorescence intensities namely signal intensities divided by corresponding areas. Cytoplasmic/Nuclear ratios were calculated by dividing mean cytoplasmic fluorescence intensities by mean nuclear fluorescence intensities.
(TIF)

S1 Table List of RIOK2 phosphorylated peptides identified and quantified by nanoLC-MS/MS analysis (see Figs 2C, S2C and S2D). Column 1: position of phosphorylated residues within RIOK2. Column 2: in bold, position of the serine residue within the sequence of the identified phosphopeptides. Column 3: global localization probability of the phosphorylated sites. Column 4 to 6: normalized intensity of RIOK2 phosphopeptides for each condition (CTL, PMA and PD+PMA) expressed as mean of biological replicates ± SD. In brackets are shown PMA/CTL and PD+PMA/CTL normalized intensity ratios. Column 7: when known, the kinases responsible for the phosphorylations are mentioned. Column 8: link to PhosphoSitePlus website providing references of studies in which each RIOK2 phosphorylated residue has already been identified (LTP: Low Throughput Publications in which modification sites were determined using methods other than discovery mass spectrometry; HTP: High Throughput Publications in which modification sites were assigned using only proteomic discovery mass spectrometry).
(DOCX)

S2 Table List of oligonucleotides, probes and plasmids used in this study. (DOCX)

S1 Data Graph numerical data. (XLSX)
