# Supplementary figures and images for: RIOK2 phosphorylation by RSK promotes synthesis of the human small ribosomal subunit
Source: PLoS Genet. 2021 Jun 14;17(6):e1009583. doi: 10.1371/journal.pgen.1009583 (PMC8224940; doi:10.1371/journal.pgen.1009583)

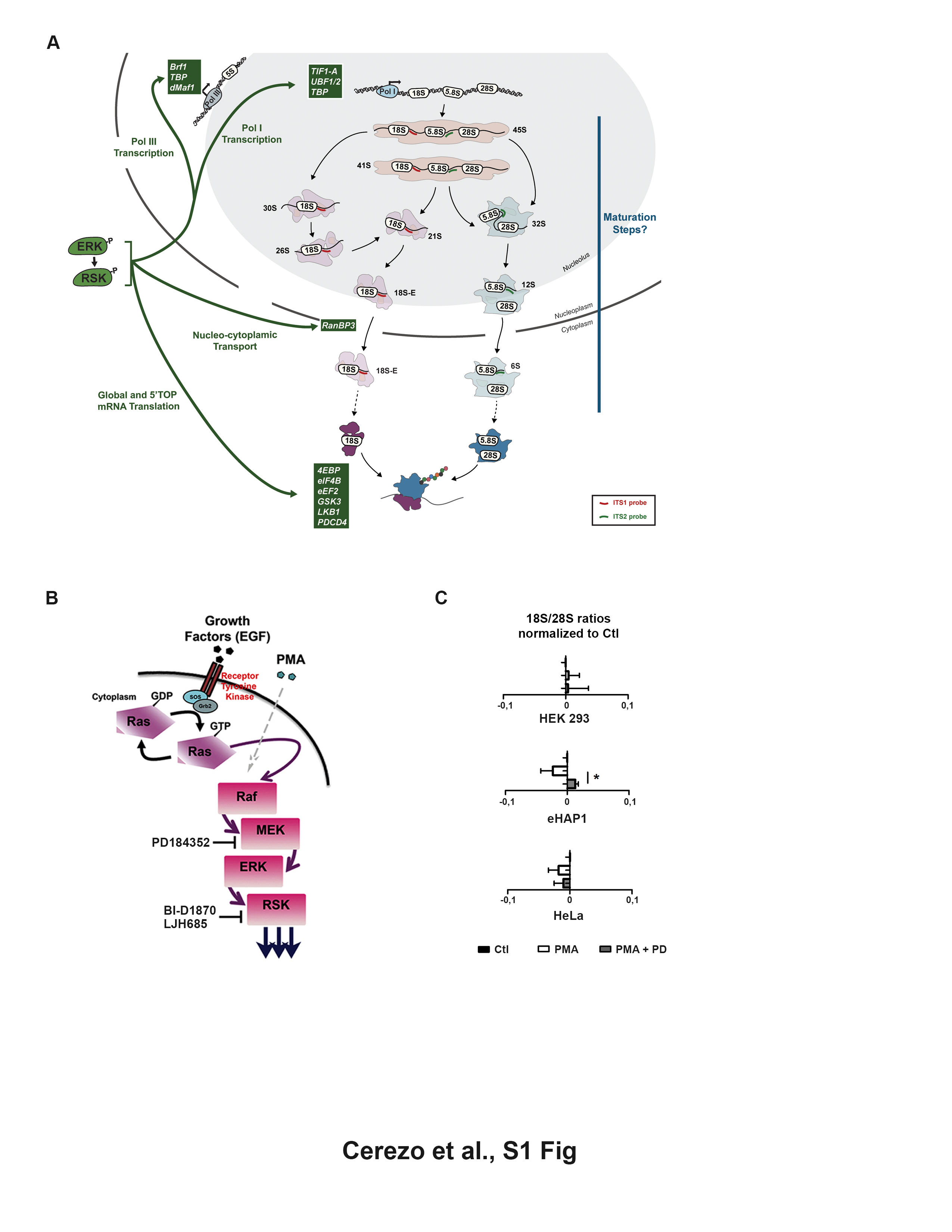

Supplement: S1 Fig — (A) Schematic representation of pre-rRNA processing in human cells, adapted from [1] with precursor subcellular localization from [64]. Position of the probes (ITS1 and ITS2) used in Northern blot (NB) experiments to detect the pre-rRNAs are indicated. The known targets of ERK and RSK kinases involved in ribosome synthesis and translation are mentioned. The MAPK pathway promotes ribosome biogenesis through the regulation of multiple stages of the process, although no pre-ribosome assembly and maturation factor has been identified so far as a direct target of ERK or RSK kinases. (B) Schematic representation of the Ras/MAPK signaling pathway with pharmacological agonists and inhibitors used in this study. (C) RAMP analyses of 18S/28S ratios detected in Fig 1A after signal quantifications using MultiGauge software (Fujifilm). (TIF) [file pgen.1009583.s001.tif]

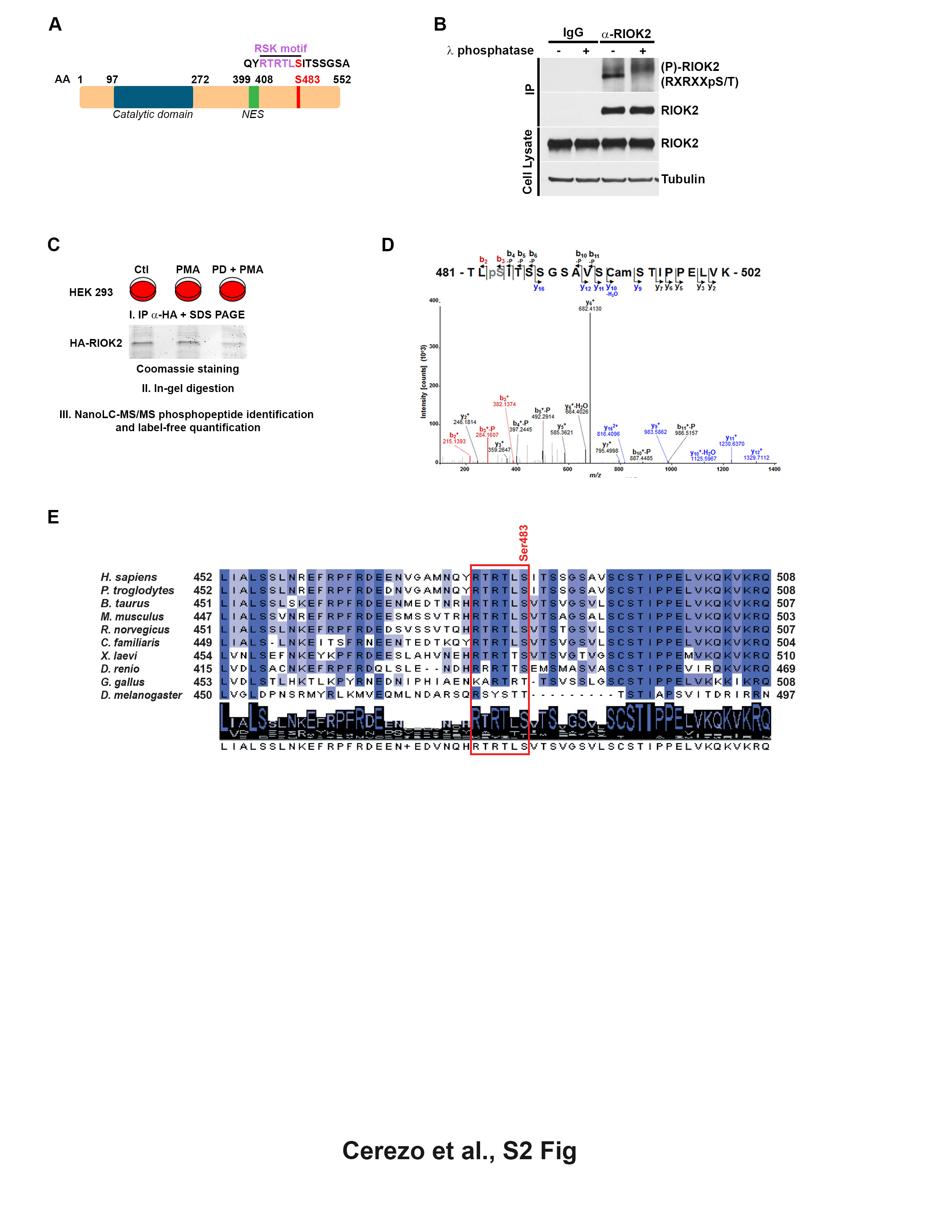

Supplement: S2 Fig — (A) Schematic location of RIOK2 Ser483 with respect to the known functional domains of the protein. (B) Endogenous RIOK2 was immunoprecipitated from serum-growing HEK293 cells using anti-RIOK2 antibodies. A control immunoprecipitation was performed in parallel using IgGs (IgG) to validate the specificity of the immunoprecipitation. After immunoprecipitation, samples were treated (+) or not (-) with λ phosphatase and analyzed by WB using the indicated antibodies. (C) HA-RIOK2 was immunoprecipitated from serum-starved (Ctl) HEK293 cells treated (PD+PMA) or not (PMA) with PD184352 (10 μM, 1h) prior to PMA stimulation (100 ng/ml, 20 min). Purified HA-RIOK2 was isolated following SDS-PAGE and Coomassie staining, in gel digested with trypsin and the resulting peptides were submitted to nano LC-MS/MS analysis. (D) HCD MS/MS spectrum of the S483 monophosphorylated peptide (doubly charged precursor ion, MH3+, at m/z 1157.5670). Highlighted in red are site-determining ions and the corresponding peaks in the spectrum. In blue are indicated fragment ions that confirm the site localization and exclude another potential site. P: loss of H3PO4 from sequence ions. pS: phosphorylated serine residue. Cam: carbamidomethylated cysteine residue. (E) Partial amino acid sequences of RIOK2 proteins from the organisms indicated on the left were aligned using Jalview 2.11.0 software and Muscle with default parameters. The bottom consensus sequence was auto-calculated. The conserved RXRXXpS motif is boxed and Ser483 of human RIOK2 is indicated. (TIF) [file pgen.1009583.s002.tif]

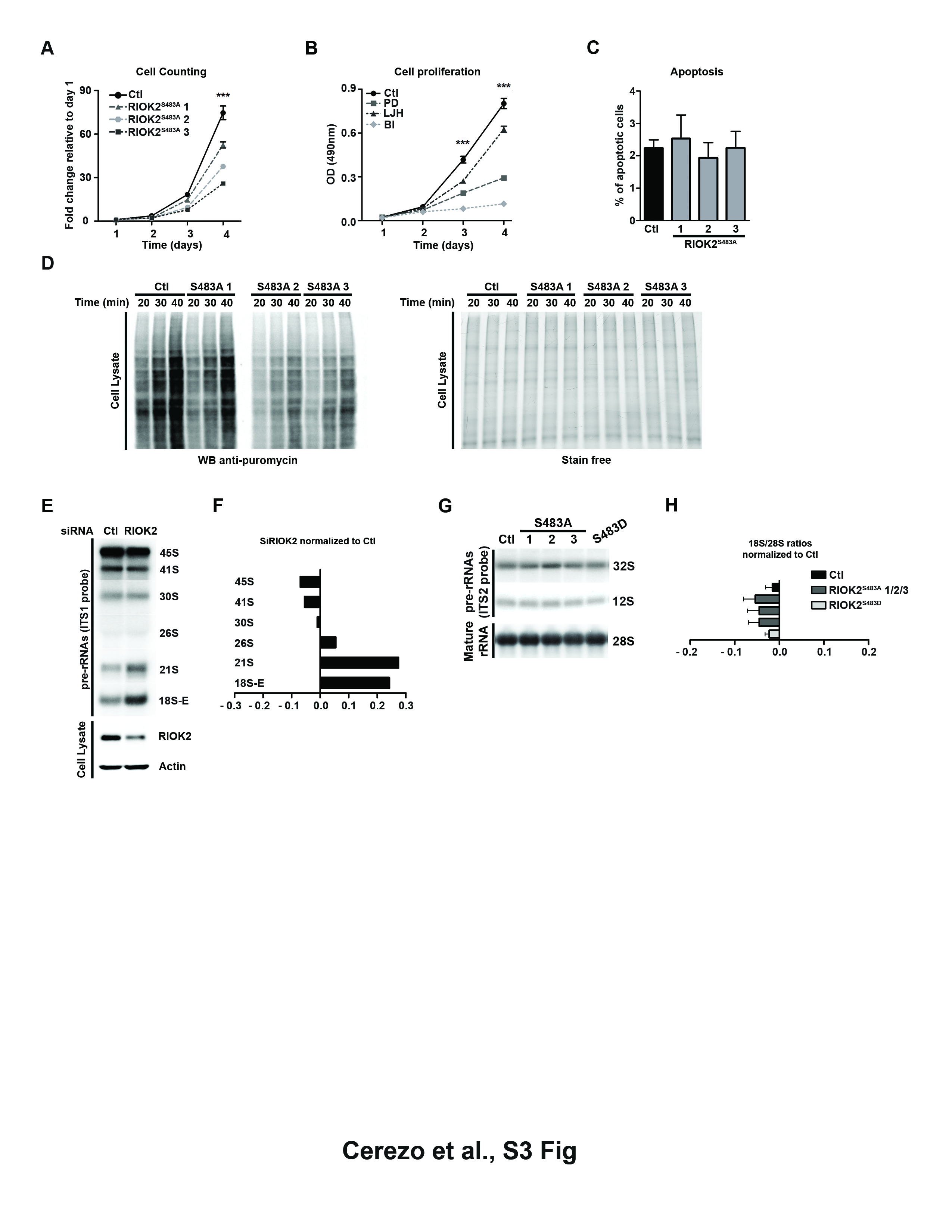

Supplement: S3 Fig — (A) Cell counting was performed on cultures of control (Ctl) and RIOK2S483A (1 to 3) eHAP1 cell lines at the indicated time points with a cell counter (Beckman-Coulter Z1). (B) MTS assays were performed on control eHAP1 cell line cultured in the presence of PD184352 (10 μM), BI-D1870 (2,5 μM), or LJH685 (10 μM). ODs at 490 nm were measured using Spectramax at the indicated time points. For (A) and (B), statistically significant differences between Ctl and other conditions are indicated by asterisks (***: P≤0.001, 2way ANOVA tests, Bonferroni posttests). (C) Apoptosis levels in cell lines from (A) were measured based on FITC-Annexin5 (FITC-A) and Propidium Iodide labelling. Apoptosis levels corresponding to FITC-A single-positive cells were monitored by FACS Verse analyzer and quantifications are expressed as percentage of total cells. No statistical difference between Ctl and RIOK2S483A cell lines was revealed (One-tailed Mann Whitney test). (D) Control (Ctl) and RIOK2S483A (1 to 3) eHAP1 cell lines were incubated with 1 μM puromycin for the indicated times. Protein extracts were analyzed by WB using anti-puromycin antibodies (left). Equal amounts of total proteins in each sample were controlled on TGX Stain-Free gels (Bio-Rad, right). (E) eHAP1 cells were transfected with siRNA targeting an irrelevant sequence (Ctl) or RIOK2. Total RNAs were extracted and pre-rRNA levels were monitored by NB. RIOK2 depletion was controlled by WB. (F) RAMP analyses of pre-rRNA levels detected in (E), as in Fig 1. (G) Total cellular RNAs were extracted from control (Ctl), RIOK2S483A or RIOK2S483D eHAP1 cell lines. Accumulation levels of large subunit pre-rRNAs (ITS2 probe) and mature 28S rRNA were analyzed by NB. (H) RAMP analyses of 18S/28S ratios detected in (G) and Fig 4C, as in Fig 1. No statistical difference between Ctl RIOK2S483A and RIOK2S483D cell lines was revealed (Kruskal-Wallis test, Dunn’s multiple comparison test) (TIF) [file pgen.1009583.s003.tif]

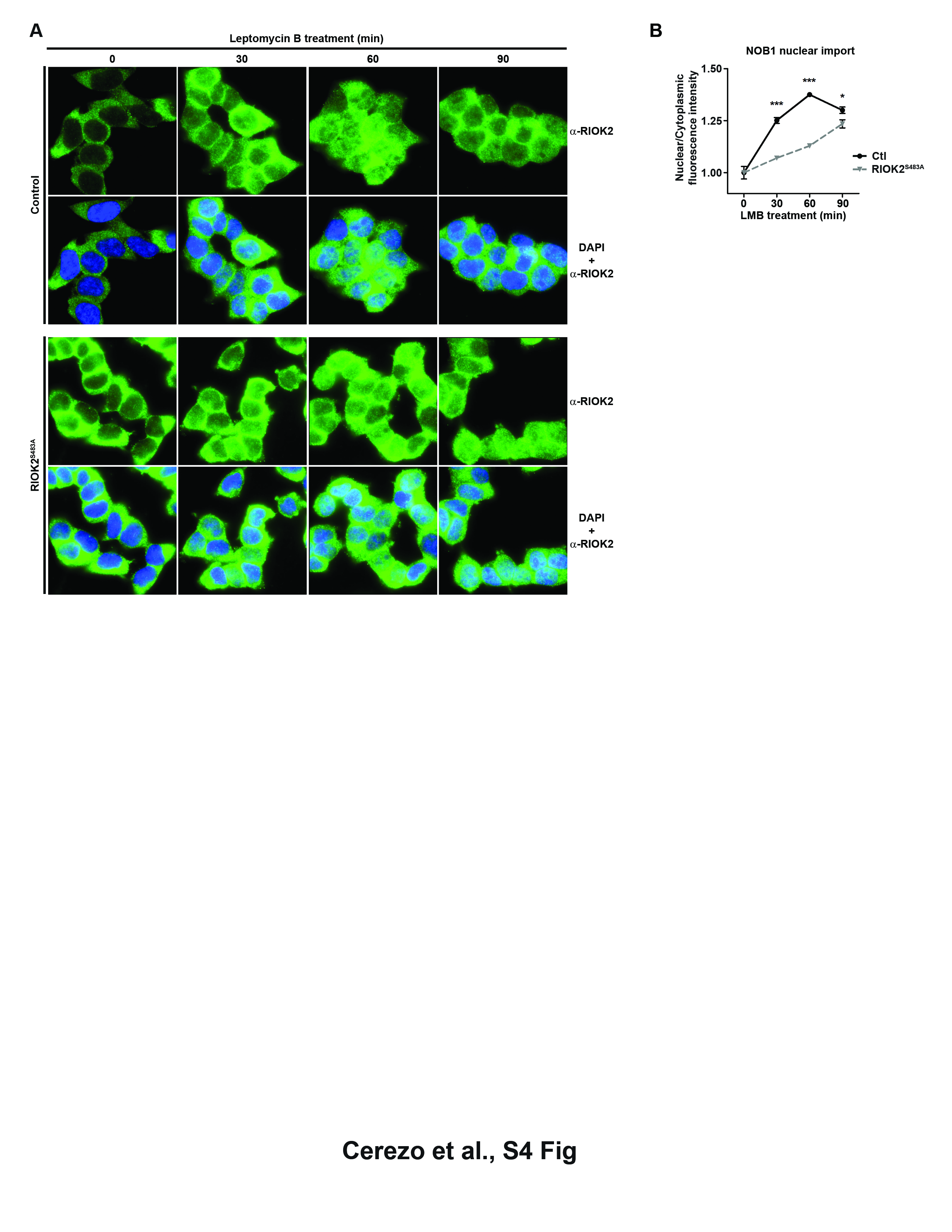

Supplement: S4 Fig — (A) RIOK2WT and RIOK2S483A eHAP1 cells were treated with Leptomycin B (LMB, 20 nM) for the indicated times. Subcellular localization of NOB1 was monitored by immunofluorescence microscopy using specific antibodies. Nuclei were visualized by DAPI staining. (B) Quantification of nuclear to cytoplasmic fluorescence ratios at the indicated time points obtained in (A) using ImageJ software (n = 100 cells from different fields), as described in Materials and Methods section and S7 Fig. Statistically significant differences are indicated by asterisks (***: P≤0.001, *: P≤0.05, 2way ANOVA tests, Bonferroni posttests). (TIF) [file pgen.1009583.s004.tif]

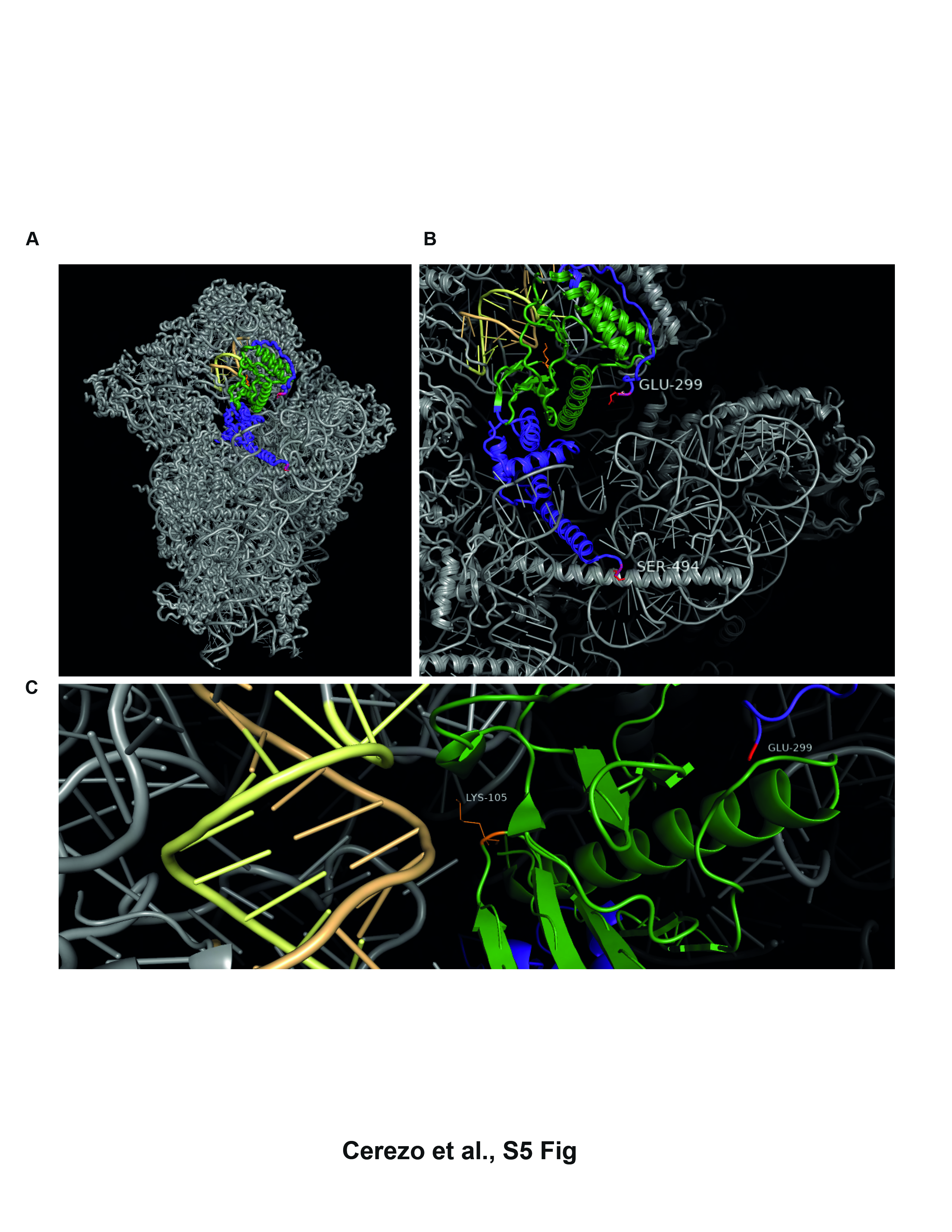

Supplement: S5 Fig — RIOK2 is coloured in purple, its catalytic domain highlighted in green and the P-loop Lys105 in orange. The protein domain comprised between Val300 to Cys493, containing Ser483, is not resolved in the structure. The flanking residues Glu299 and Ser494 are coloured in red. Helix 80 of the 18S rRNA (residues 1226–1237 and 1520–1532) has been coloured in yellow. (A) Global view of RIOK2-bound pre-40S particle. (B) Closer view of RIOK2 with the residues flanking the domain containing Ser483 (not resolved) at the surface of the particle. (C) Closer view of the catalytic domain showing that catalytic P-loop lysine 105 is in close contact to helix 30 of the 18S rRNA. (TIF) [file pgen.1009583.s005.tif]

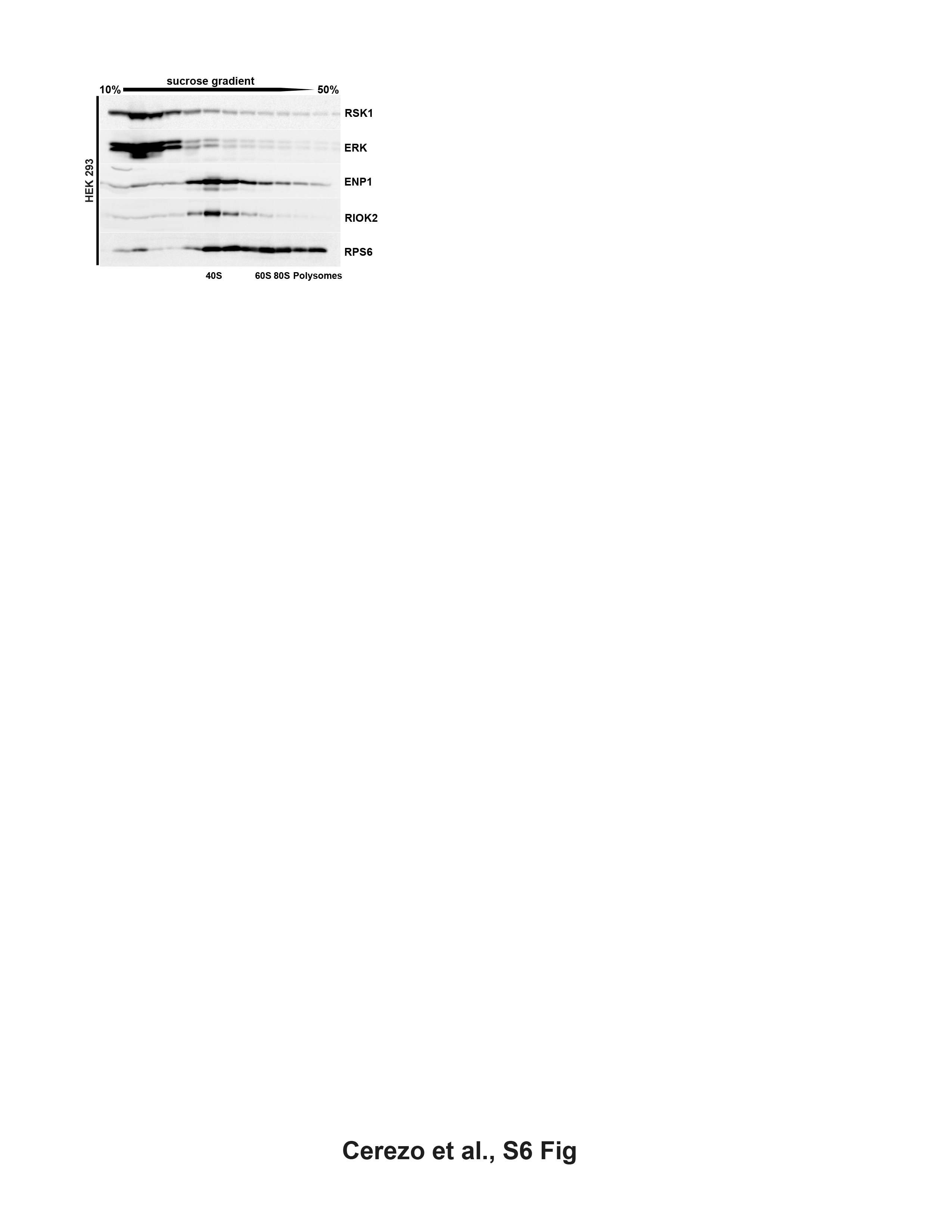

Supplement: S6 Fig — Proteins were TCA-precipitated from collected fractions and the proteins indicated on the right were detected by WB. (TIF) [file pgen.1009583.s006.tif]

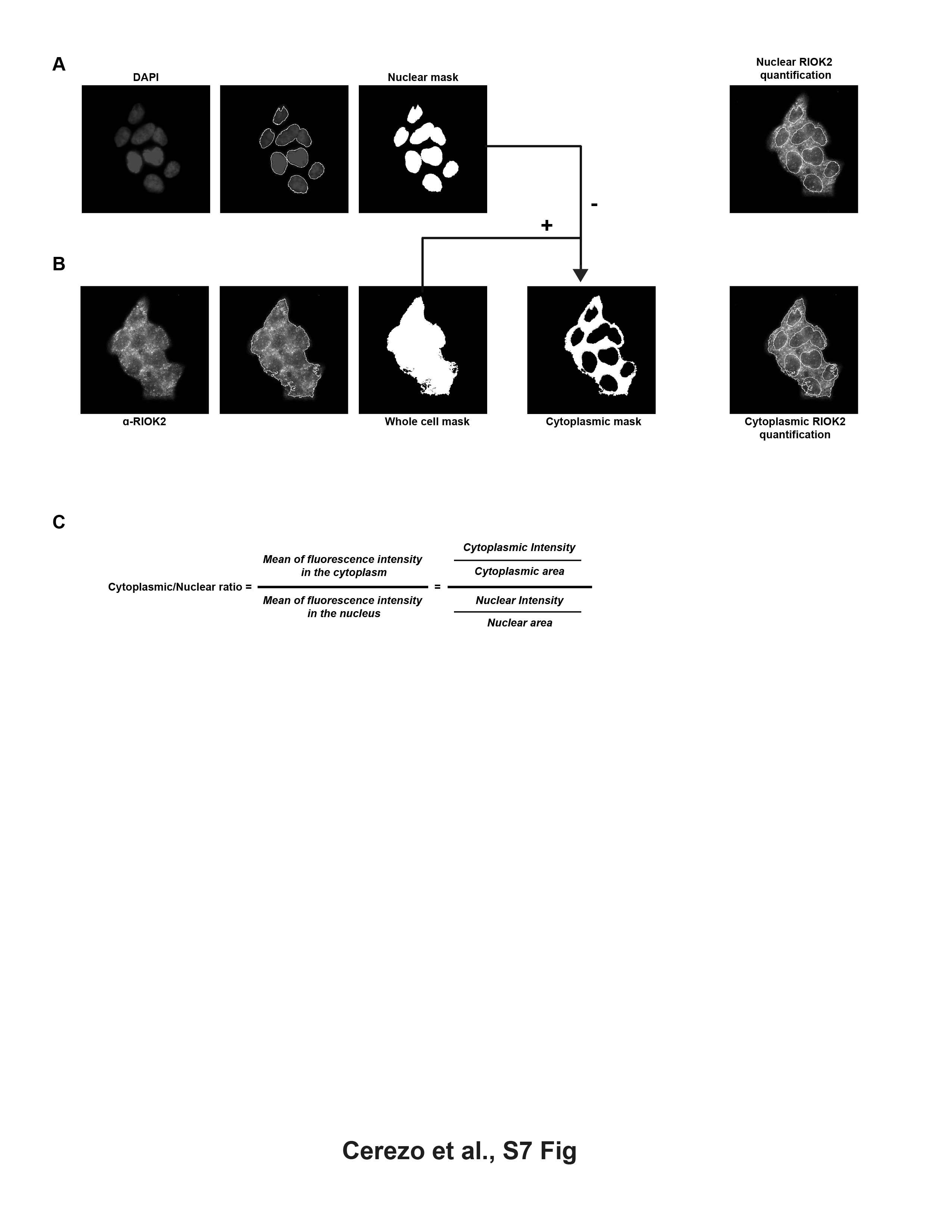

Supplement: S7 Fig — RIOK2 nuclear and cytoplasmic signals have been quantified with ImageJ software as follows. (A) Nuclear masks (binary images) were generated from DAPI signal intensities and transposed to the corresponding α-RIOK2 immunofluorescence images to quantify the nuclear intensities. (B) To quantify α-RIOK2 cytoplasmic intensities, cytoplasmic masks were obtained by subtracting nuclear masks intensities to whole cell α-RIOK2 signals. (C) All signals were expressed as mean of fluorescence intensities namely signal intensities divided by corresponding areas. Cytoplasmic/Nuclear ratios were calculated by dividing mean cytoplasmic fluorescence intensities by mean nuclear fluorescence intensities. (TIF) [file pgen.1009583.s007.tif]
