# Supplementary material for: RIOK2 phosphorylation by RSK promotes synthesis of the human small ribosomal subunit
Source: PLoS Genet. 2021 Jun 14;17(6):e1009583. doi: 10.1371/journal.pgen.1009583 (PMC8224940; doi:10.1371/journal.pgen.1009583)
Supplement: S1 Table — Column 1: position of phosphorylated residues within RIOK2. Column 2: in bold, position of the serine residue within the sequence of the identified phosphopeptides. Column 3: global localization probability of the phosphorylated sites. Column 4 to 6: normalized intensity of RIOK2 phosphopeptides for each condition (CTL, PMA and PD+PMA) expressed as mean of biological replicates ± SD. In brackets are shown PMA/CTL and PD+PMA/CTL normalized intensity ratios. Column 7: when known, the kinases responsible for the phosphorylations are mentioned. Column 8: link to PhosphoSitePlus website providing references of studies in which each RIOK2 phosphorylated residue has already been identified (LTP: Low Throughput Publications in which modification sites were determined using methods other than discovery mass spectrometry; HTP: High Throughput Publications in which modification sites were assigned using only proteomic discovery mass spectrometry). (DOCX) [file pgen.1009583.s008.docx]

**Cerezo et al., S1 Table**

| **Phospho-residue** | **Phosphopeptide sequence** | **Global localization probability (%)** | **Normalized intensity ± SD** | | | **Kinase** | **PhosphoSitePlus Resource** |
| --- | --- | --- | --- | --- | --- | --- | --- |
|  |  |  | **CTL** | **PMA**  (PMA/CTL ratio) | **PD+PMA**  (PD+PMA/CTL ratio) |  |  |
| **S332** | EG**S**EFSFSDGEVAEK | **99** | 1.96E+07 ± 4.77E+06 | 3.55E+07 ± 3.13E+07  **(1.81)** | 2.21E+07 ± 1.24E+07  **(1.13)** | - | **HTP:** <https://www.phosphosite.org/siteGroupAction.action?id=470784&protOrg=2360&showAllSites=true&showHTPRefsOnly=true> |
| **S335** | EGSEF**S**FSDGEVAEK | **62** | 1.43E+07 | 2.19E+07  **(1.53)** | 2.99E+07  **(2.09)** | PLK1 | **LTP:** <https://www.phosphosite.org/siteGroupAction.action?id=471042&protOrg=2360&showAllSites=true&showLTPRefsOnly=true>  **HTP:** <https://www.phosphosite.org/siteGroupAction.action?id=471042&protOrg=2360&showAllSites=true&showHTPRefsOnly=true> |
| **S337** | EGSEFSF**S**DGEVAEK | **83** | 2.26E+07 ± 6.57E+06 | 3.06E+07 ± 2.27E+07  **(1.35)** | 2.45E+07 ± 1.50E+07  **(1.08)** | - | **HTP:** <https://www.phosphosite.org/siteGroupAction.action?id=470785&protOrg=2360&showAllSites=true&showHTPRefsOnly=true> |
| **S350** | AEVYG**S**ENESER | **97** | 2.18E+08 ± 2.39E+07 | 2.52E+08 ± 9.29E+07  **(1.15)** | 2.41E+08 ± 1.07E+08  **(1.10)** | - | **LTP:** <https://www.phosphosite.org/siteGroupAction.action?id=3211074&protOrg=2360&showAllSites=true&showLTPRefsOnly=true>  **HTP:** <https://www.phosphosite.org/siteGroupAction.action?id=3211074&protOrg=2360&showAllSites=true&showHTPRefsOnly=true> |
| **S354** | AEVYGSENE**S**ER | **61** | 1.31E+06 ± 3.50E+05 | 1.85E+06 ± 3.15E+05  **(1.41)** | 2.72E+06 ± 8.84E+04  **(2.09)** | - | **HTP:** <https://www.phosphosite.org/siteGroupAction.action?id=36127122&protOrg=2360&showAllSites=true&showHTPRefsOnly=true> |
| **S369** | **S**SGDPEQIKEDSLSEESADAR | **54** | 3.43E+07 ± 1.82E+07 | 7.13E+07 ± 5.30E+07  **(2.08)** | 5.09E+07 ± 3.49E+07  **(1.49)** | - | **HTP:** <https://www.phosphosite.org/siteGroupAction.action?id=56670380&protOrg=2360&showAllSites=true&showHTPRefsOnly=true> |
| **S380** | SSGDPEQIKED**S**LSEESADAR | **85** | 1.16E+07 ± 3.89E+06 | 2.60E+07 ± 1.35E+07  **(2.24)** | 1.79E+07 ± 1.03E+07  **(1.54)** | PLK1 | **LTP:** <https://www.phosphosite.org/siteGroupAction.action?id=3211080&protOrg=2360&showAllSites=true&showLTPRefsOnly=true>  **HTP:** <https://www.phosphosite.org/siteGroupAction.action?id=3211080&protOrg=2360&showAllSites=true&showHTPRefsOnly=true> |
| **S382** | SSGDPEQIKEDSL**S**EESADAR | **75** | 9.38E+06 ± 2.07E+06 | 6.55E+06 ± 3.93E+06  **(0.70)** | 8.36E+06 ± 9.31E+06  **(0.89)** | - | **HTP:** <https://www.phosphosite.org/siteGroupAction.action?id=3211083&protOrg=2360&showAllSites=true&showHTPRefsOnly=true> |
| **S390** | **S**FEMTEFNQALEEIK | **97** | 2.15E+07 ± 2.66E+06 | 1.53E+07 ± 9.21E+06  **(0.71)** | 2.27E+07 ± 1.28E+07  **(1.05)** | - | **HTP:** <https://www.phosphosite.org/siteGroupAction.action?id=4270708&protOrg=2360&showAllSites=true&showHTPRefsOnly=true> |
| **S412** | GQVVENN**S**VTEFSEEK | **89** | 1.12E+07 ± 3.26E+06 | 8.18E+06 ± 2.09E+06  **(0.73)** | 6.58E+06 ± 3.53E+06  **(0.59)** | - | **HTP:** <https://www.phosphosite.org/siteGroupAction.action?id=15422926&protOrg=2360&showAllSites=true&showHTPRefsOnly=true> |
| **S442** | VQGGVPAG**S**DEYEDECPHLIALSSLNR | **97** | 5.23E+07 ± 1.06E+07 | 5.56E+07 ± 2.33E+07  **(1.06)** | 5.23E+07± 2.43E+07  **(1.00)** | - | **HTP:** <https://www.phosphosite.org/siteGroupAction.action?id=1211754&protOrg=2360&showAllSites=true&showHTPRefsOnly=true> |
| **S483** | TL**S**ITSSGSAVSCSTIPPELVK | **58** | 4.09E+07 ± 7.78E+06 | 2.21E+08 ± 4.62E+07  **(5.40)** | 5.20E+07 ± 1.22E+07  **(1.27)** | RSK | **HTP:** <https://www.phosphosite.org/siteGroupAction.action?id=7446950&protOrg=2360&showAllSites=true&showHTPRefsOnly=true> |
