# Supplementary material for: RIOK2 phosphorylation by RSK promotes synthesis of the human small ribosomal subunit
Source: PLoS Genet. 2021 Jun 14;17(6):e1009583. doi: 10.1371/journal.pgen.1009583 (PMC8224940; doi:10.1371/journal.pgen.1009583)
Supplement: S2 Table — (DOCX) [file pgen.1009583.s009.docx]

**Cerezo et al., S2 Table**

| **Oligonucleotides** | **Source** |
| --- | --- |
| FW primer for cloning in plasmid 86613 (RIOK2 gRNA):  5’-CACCGACTCTGAGTATCACTTCTTC-3’ | This paper |
| RV primer for cloning in plasmid 86613 (RIOK2 gRNA):  5’-AAACGAAGAAGTGATACTCAGAGTC-3’ | This paper |
| Donor template for RIOK2^S483A^ point mutation:  5’-TTTTAGAGATGAAGAAAATGTGGGAGCTATGAATCAGTATAGAACAAGAACTCTGGCCATCACTTCTAGCGGCAGTGCTGTAAGCTGTTCAACAATTCCTCCAGTAAGTAGTCATTCAACATGTA-3’ | This paper |
| Donor template for RIOK2^S483D^ point mutation:  5’-TTTTAGAGATGAAGAAAATGTGGGAGCTATGAATCAGTATAGAACAAGAACTCTGGATATCACTTCTAGCGGCAGTGCTGTAAGCTGTTCAACAATTCCTCCAGTAAGTAGTCATTCAACATGTA-3’ | This paper |
| Donor template for ouabain resistance:  5’-CAATGTTACTGTGGATTGGAGCGATTCTTTGTTTCTTGGCTTATAGCATCAGAGCTGCTACAGAAGAGGAACCTCAAAACGATGACGTGAGTTCTGTAATTCAGCATATCGATTTGTAGTACACATCAGATATCTT-3’ | [1] |
| FW primer for PCR amplification of RIOK2 S483 locus:  5’- GGAGGTGATAAATTTCAATGGC-3’ | This paper |
| RV primer for PCR amplification of RIOK2 S483 locus:  5’- AACTCTACCACATCCTTTGAGC-3’ | This paper |
| FW primer for RIOK2 cDNA cloning into pCMV-3HA:  5’-CCGGATCCCCGTCGAATGGGGAAAGTGAATGTGG-3’ | This paper |
| RV primer for RIOK2 cDNA cloning into pCMV-3HA:  5’-CCGGGGTACCGTCGATTATTCTCCCCAAAAGCTAG-3’ | This paper |
| FW primer for RIOK2 cDNA cloning into pCMV-3FLAG: | This paper |
| RV primer for RIOK2 cDNA cloning into pCMV-3FLAG: | This paper |
| FW primer for NOB1 cDNA cloning into pCMV-3HA:  5’-CCGGATCCCCGTCGAATGGCTCCAGTGGAGCAC-3’ | This paper |
| RV primer for NOB1 cDNA cloning into pCMV-3HA:  5’-CCGGGGTACCGTCGATCACCTTTTCTTCACAAACTT-3’ | This paper |
| FW primer for S483A mutagenesis in pCMV-3HA-RIOK2 and pCMV-3FLAG-RIOK2:  5’-TCAGTATAGAACAAGAACTCTGGCCATCACTTCTTCAGGCAGTGCTG-3’ | This paper |
| RV primer for S483A mutagenesis in pCMV-3HA-RIOK2 and pCMV-3FLAG-RIOK2:  5’-CAGCACTGCCTGAAGAAGTGATGGCCAGAGTTCTTGTTCTATACTGA | This paper |
| FW primer for T481A mutagenesis in pCMV-3HA-RIOK2:  5’-CTATGAATCAGTATAGAACAAGAGCCCTGAGTATCACTTCTTCAGGCAG-3’ | This paper |
| RV primer for T481A mutagenesis in pCMV-3HA-RIOK2:  5’-CTGCCTGAAGAAGTGATACTCAGGGCTCTTGTTCTATACTGATTCATAG-3’ | This paper |
| FW primer for T/S2A mutagenesis in pCMV-3HA-RIOK2:  5’-TGAATCAGTATAGAACAAGAGCCCTGGCCATCACTTCTTCAGG-3' | This paper |
| RV primer for T/S2A mutagenesis in pCMV-3HA-RIOK2:  5’-CCTGAAGAAGTGATGGCCAGGGCTCTTGTTCTATACTGATTCA-3’ | This paper |
| FW primer for S483D mutagenesis in pCMV-3FLAG-RIOK2:  5’-AACTCTGGATATCACTTCTTCAGGCAGTGC-3’ | This paper |
| RV primer for S483D mutagenesis in pCMV-3FLAG-RIOK2:  5’-GTGATATCCAGAGTTCTTGTTCTATACTGATTC-3’ | This paper |
| FW primer for cloning of WT or S483A RIOK2 C-ter domain (D443-E552) into pGEX-4T-1:  5’-TGGATCCCCGGAATTCGACGAGTATGAAGATGAATG -3’ | This paper |
| RV primer for cloning of WT or S483A RIOK2 C-ter domain (D443-E552) into pGEX-4T-1:  5’-GTCGACCCGGGAATTCTTATTCTCCCCAAAAGCTGG-3' | This paper |

| **Probes** | **Source** |
| --- | --- |
| Northern blot probe to detect SSU pre-rRNAs:  5’-CCTCGCCCTCCGGGCTCCGTTAATGATC-3’ | [2] |
| FISH probe to detect SSU pre-rRNAs:  5’-CCT*CGCCCTCCGGGCT*CCGTTAATGAT*C-3’  T*: C6-amino-modified desoxythymidines conjugated to Cy5. | [2] |
| Northern blot probe to detect LSU pre-rRNAs:  5’-GCGCGACGGCGGACGACACCGCGGCGTC-3’ | [2] |
| Northern blot probe to detect mature 18S rRNA::  5’-TTTACTTCCTCTAGATAGTCAAGTTCGACC-3’ | [3] |
| Northern blot probe to detect mature 28S rRNA:  5’CCCGTTCCCTTGGCTGTGGTTTCGCTAGATA-3’ | [3] |
| siRNA FW sequence against RSK1:  5’-CCCAACATCATCACTCTGA-3’ | This paper |
| siRNA FW sequence against RSK2:  5’-CCCAACATCATCACTCTGA-3’ | This paper |

| **Plasmids** | **Source** | **Identifier** |
| --- | --- | --- |
| 86613 plasmid (eSpCas9(1.1)_No_FLAG_ATP1A1_G3_Dual_sgRNA) | Addgene | Cat#86613 |
| 86613-RIOK2-gRNA plasmid | This paper | N/A |
| pCMV-3HA (5’ tagging) | D. Trouche Lab | N/A |
| p3xFLAG-CMV-10 (5’ tagging) | Merck | Cat#E7658 |
| pGEX-4T-1 | GE Healthcare | Cat#GE28-9545-49 |
| pCMV-3HA-RIOK2 | This paper | N/A |
| pCMV-3HA-RIOK2^S483A^ | This paper | N/A |
| pCMV-3HA-RIOK2^T481A^ | This paper | N/A |
| pCMV-3HA-RIOK2^T/S2A^ | This paper | N/A |
| pCMV-3HA-NOB1 | This paper | N/A |
| pKH3-RSK1 | [4] | N/A |
| pKH3-RSK2 | [4] | N/A |
| pKH3-RSK3 | [4] | N/A |
| pKH3-RSK4 | [4] | [4][4]N/A |
| pCMV-3FLAG-RIOK2 | This paper | N/A |
| pCMV-3FLAG-RIOK2^S483A^ | This paper | N/A |
| pCMV-3FLAG-RIOK2^S483D^ | This paper | N/A |
| pGEX-4T-1-RIOK2-Cter (D443-E552) | This paper | N/A |
| pGEX-4T-1-RIOK2^S483A^-Cter (D443-E552) | This paper | N/A |
| ShRSK1vector TRCN470 | Merck | Mission TRC shRNA # TRCN470 |
| ShRSK2 vector TRCN537 | Merck | Mission TRC shRNA # TRCN537 |

**References**

**1. Agudelo D, Duringer A, Bozoyan L, Huard CC, Carter S, Loehr J, et al. Marker-free coselection for CRISPR-driven genome editing in human cells. Nat Methods. 2017;14: 615–620. doi:10.1038/nmeth.4265**

**2. Rouquette J, Choesmel V, Gleizes P-E. Nuclear export and cytoplasmic processing of precursors to the 40S ribosomal subunits in mammalian cells. EMBO J. 2005;24: 2862–2872. doi:10.1038/sj.emboj.7600752**

**3. Bonnart C, Gérus M, Hoareau-Aveilla C, Kiss T, Caizergues-Ferrer M, Henry Y, et al. Mammalian HCA66 protein is required for both ribosome synthesis and centriole duplication. Nucleic Acids Res. 2012;40: 6270–6289. doi:10.1093/nar/gks234**

**4. Roux PP, Richards SA, Blenis J. Phosphorylation of p90 ribosomal S6 kinase (RSK) regulates extracellular signal-regulated kinase docking and RSK activity. Mol Cell Biol. 2003;23: 4796–4804. doi:10.1128/mcb.23.14.4796-4804.2003**
