# Supplementary material for: RIOK2 phosphorylation by RSK promotes synthesis of the human small ribosomal subunit
Source: PLoS Genet. 2021 Jun 14;17(6):e1009583. doi: 10.1371/journal.pgen.1009583 (PMC8224940; doi:10.1371/journal.pgen.1009583)
Supplement: Attachment — Submitted filename: Cerezo et al, Response to Reviewers.pdf [file pgen.1009583.s011.pdf]

Point-by-point response to the Reviewers' comments:

We thank the referees for their feedback and constructive comments to our work. We provide here a point-by-point response (text in green) to the comments of Reviewers #1, #2 and #3 (text in grey and italic). The corresponding modifications to the text are indicated in red in the word file of the manuscript.

Reviewer #1 (Evidence, reproducibility and clarity (Required)):

The manuscript addresses an important topic, the posttranscriptional maturation of ribosomes. This topic is inherently interesting because we normally think of ribosome biogenesis as a sequential series of steps that automatically proceeds and cannot be "accelerated" in physiological conditions, but only "delayed" in the presence of genetic mutations. In short, the manuscript proposes that RIOK2 phosphorylation by the action of RSK, below the Ras/MAPK pathway promotes the synthesis of the human small ribosomal subunit.

I honestly admit that I have some difficulties in reviewing this manuscript. The quality of the presented data is, in generally, good. However, overall I find the whole manuscript preliminary and I am not much convinced of the conclusions. Several aspects are superficially analyzed. In short, I think that most of the conclusions are not fully supported by the data because shortcuts are present. A list of all the aspects that I found wrong are listed.

Biological issue

1. The authors claim that the effects of the inhibition of the maturation of ribosomes by acting on a pathway upstream of RIOk2 are limited to the 40S subunit. This is far from being a trivial point, for the following reason. RIOK2 is known to affect the maturation of 40S ribosomes. Hence, the fact that using an upstream inhibitor of the MAPK pathway such as PD does not inhibit 60S processing in reality would argue against a biologically relevant control in ribosome maturation (of the MAPK patheay). Have the authors considered this? In a way, also, given the fact that the mutants confirm a role in 18S final maturation, it is a bit complex to put all the data in a clear biological context.

We agree that we put more emphasis on the effects on the pre-40S pathway than on the pre-60S pathway in the original manuscript but we did not claim that the effects of PD or LJH inhibitors of the MAPK pathway are restricted to the 40S subunit. We described that the effect of PD or LJH on the 32S was less severe than on the 30S, and we did mention variations of the 12S intermediate, which is a precursor to the 5.8S rRNA in pre-60S particles. These changes are in the same range of amplitude as the changes in the 21S and 18S-E intermediates in the small subunit pathway. The Northern blot data concerning the pre-60S pathway were placed in the supplementary material of the original manuscript, which may have left the reader with an impression of lesser emphasis. We rephrased this part in the present revised version of the manuscript (Page 6, Line 15) and we now show the pre-40S and pre-60S intermediates on the same figures (Figures 1A and 1B).

In addition, we complemented these data with pulse-chase labeling experiments and provide a more dynamic analysis of the pre-rRNA processing defects resulting from inactivation of the MAPK pathway (Figures 1C and 1D). These data show that production of both the 18S rRNA and 28S rRNAs are delayed upon inhibition of the MAPK pathway. Furthermore, as requested by Reviewer #2 (see below), we have systematically quantified more accurately all the Northern blot data using the RAMP analysis (Ratio Analysis of Multiple Precursors). Altogether, these data strengthen the conclusion that the MAPK pathway participates in the regulation of both the pre-40S and pre-60S maturation pathways.

A number of specific issues will be concisely described.

Manuscript very well written. Data do not always support the strong conclusions. Low magnitude of the observed effects.

In introduction the authors make a general claim that ribosome biogenesis is one of the most energetically demanding cellular activities. This statement lingers in the literature since 15 years but in

reality it has never been formally proved for mammalian cells, and certainly not for HEK293 cells. The original statement, to my knowledge, can be traced by some obscure statement referred to the yeast case and then repeated as a truth. In conclusion, besides being a very banal observation, it should be referenced.

We agree with this comment of Reviewer #1. The original statement has been proposed by Jonathan R. Warner (Warner, 1999, TiBS and references therein) and data from the Bähler group also supported this statement (Marguerat et al., 2012, Cell). However, these data were indeed referring to yeast (*S. cerevisiae* and *S. pombe*). In the revised version of the manuscript, we introduced the reference of a review providing quantitative data of ribosome biogenesis in human cells (Lewis & Tollervey, 2000, Science) and we modified the problematic sentence as follows:" *Growing human cells produce around 7500 ribosomal subunits per minutes (Lewis and Tollervey 2000), which represents a significant expenditure of energy.*" (Page 3, Line 25).

Growth factors, energy status are not cues but are proteins or metabolites (introduction).

We agree with this comment of Reviewer #1. We changed the text accordingly in the revised version of the manuscript (Page 4, Line 4).

Authors write about mTOR without making statements on mTORC1/2. This is very obsolete. Also I am not sure that the choice of Geyer et al., 1982, and subsequent papers makes much sense. At the very minimum TOP mRNA concepts and mTORC1 must be defined.

We provide more details on the mTOR pathway in the revised version of the manuscript according to this suggestions (Page 4, Lines 8 and 22).

The authors claim that their work fills a major gap between known functions of MAPK and cytoplasmic translation. I would not be so sure about it.

Our original sentence stated that "*our work fills a major gap between currently known functions of MAPK signaling in Pol I transcription and cytoplasmic translation*". Indeed, although MAPK signaling was known to regulate Pol I transcription and cytoplasmic translation, the impact of the pathway on the post-transcriptional steps of ribosome synthesis, namely pre-ribosome assembly and maturation, has been very little investigated and remains poorly understood. Our data provides the first example of a detailed mechanism of regulation of the maturation of pre-ribosomal particles by the MAPK pathway. Reviewers #2 and #3 seem to agree with this point:

Reviewer #2: "*However, there is a lacking mechanistic connection of signaling pathways to pre-rRNA processing and maturation steps of ribosome biogenesis. The authors set out to provide a specific example of a direct target of MAPK signaling, RSK that regulates pre-rRNA maturation through the phosphorylation of a ribosome assembly factor (RIOK2), offering for the first time providing mechanistic insight into MAPK regulation of pre-rRNA maturation.*"

Reviewer #3: "*With these provisos, the work is technically good and will be of considerable interest to the field. The post-transcriptional regulation of ribosome synthesis is increasingly recognized a significant topic.*"

Results. Authors start with a major mistake, i.e. that PMA selectively stimulates the MAPK pathway. Perhaps it stimulates, certainly it does not do it selectively.

We agree with this comment. We removed the term "selectively" in the problematic sentence (Page 6, Line 8).

RIOK2 phosphosites are first found by bioinformatics analysis. It should be noted that the predicted phosphosite (S483) is found only in a limited set of datasets from MS databases. The actual importance of this site would not emerge from unbiased studies. Also, there are many other phosphosites that were not analyzed in this study.

We agree with Reviewer #1 that phosphorylation of S483 of RIOK2 has been detected in a limited number of mass spectrometry datasets, but these datasets have been reported in high impact journals (Nature Methods, Mol Cell Proteomics, Science), attesting of the quality of these studies.

As mentioned by Reviewer #1, there are several other phosphosites within RIOK2 that were not analyzed in our study. We provided the list of these phosphosites in Supplemental Table S1 of the original manuscript. Besides T481 and S483, none of the other sites belong to consensus motifs recognized by ERK or RSK at medium and high stringency. They are therefore less relevant to our study. We only analyzed phosphorylation at S483 because: (i) our mass spectrometry analysis revealed that S483 is the only phosphosite in RIOK2 whose level increases upon MAPK activation but not in the presence of the MAPK inhibitor PD184352 (Figure 2C); (ii) our in vitro kinase assay showed that the phosphorylation level of RIOK2 by RSK is residual when S483 is replaced by a non-phosphorylatable alanine (Figure 3G); (iii) our data presented in Figure 2D further show that mutation of T481 to an alanine does not prevent RIOK2 phosphorylation on RxRxxS/T motifs upon stimulation of the MAPK pathway.

We clarified this point in the relevant part of the result section of the revised version of the manuscript (Page 7, Lines 18 and 25, Page 8, Line 26 and Page 9, Line 6).

Throughout the paper the authors use the word strongly, significantly, but the actual effects seem in general quite marginal.

We agree with Reviewer #1 that some of the phenotypes described in the manuscript are modest, in particular the phenotypes resulting from the S483A mutation of RIOK2, which is not aberrant for a point mutation. We rephrased several sentences throughout the manuscript to soften the formulations in the description and interpretation of the data and in the conclusions.

Discussion. The authors claim that they provide solid evidence on MAPK signalling to ribosome maturation. At the very best this is circumstantial evidence for the 40S maturation.

We rephrased the sentence accordingly (Page 17, Line 5): "*Our study provides evidence that MAPK signaling applies another level of coordination during ribosome biogenesis, by directly regulating pre-40S particle assembly and maturation.*"

Figure 1.

Unclear why LJH should increase P-ERK.

A negative feedback loop has been described in the MAPK pathway whereby RSK activation partially inhibits ERK phosphorylation (Saha *et al.,* 2012, Horm Metab Res; Dufresne *et al.,* 2001, MCB; Schneider *et al.,* 2011, Neurochem; Nett *et al.*, 2018, EMBO Rep). Inactivation of RSK with LJH alleviates this inhibition, which results in increased phosphorylation levels of ERK. We added this information in the revised version of the manuscript along with the corresponding references (Page 10, Line 23).

General lack of quantitation (sd, replicates, bars). Experiment done only on a single cell line in a single experimental setup.

As also requested by Reviewer #2 (Major comment 1), we applied RAMP quantifications systematically to all Northern blot data in the revised version of the manuscript. We included error bars corresponding to biological replicates (n=3 in the large majority of the experiments).

Furthermore, in order to validate the impact of the MAPK pathway on pre-ribosome assembly and maturation in different cell lines, we performed the same experiments using PD inhibitors in three human cell lines (HEK293, eHAP1 and HeLa) and provided in the revised version of the manuscript a figure with accurate RAMP quantifications, error bars and statistical significance (Figure 1). Although some variations were observed from cell line to cell line, MAPK inhibition systematically induced an accumulation of the early 30S precursor of the pre-40S pathway and a depletion of all downstream intermediates in all cell lines as well as a reduction in the precursors of the pre-60S particles (32S and 12S).

Very different effects on 21S by LJH, PMA and siRNA for RIOK2. Overall the message given by the authors is to me mysterious.

We assume that the reviewer wanted to point out the difference between PMA, PMA+LJH and **shRNAs** for **RSK** since we did not perform RNAi targeting RIOK2 in the experiments shown in figure 1. We agree with this comment. We believe that this difference is likely due to experimental setups that are different between both experiments. In the experiment using inhibitors, we assessed short-term effects of RSK inhibition after acute stimulation of the MAPK pathway (starved cells stimulated with PMA), while in the experiment using shRSK, we monitored long term effects of RSK depletion in serum-growing cells in which other signaling pathways are also active. Prolonged RSK depletion is likely to induce pleiotropic cellular effects, which would interfere with ribosome biogenesis both directly and indirectly. These differences probably explain the variable effects on the 21S intermediate. However, in both experiments we do observe an accumulation of the early 30S intermediate, consistent with the phenotype observed when ERK is inactivated (PD inhibitor), therefore indicating that RSK regulates some post-transcriptional stages of ribosome biogenesis.

To make our message clearer, we have replaced the experiments using shRSK and RSK inhibitors with a more detailed analysis of the role of the MAPK pathway in the maturation of pre-ribosomal particles, including a metabolic labeling analysis and the use of different cell lines, as mentioned above (Figure 1).

Figure 2.

Several red flags. For instance in 2C the loaded levels of RIOK2-HA loaded are clearly less than the ones of the other genotypes, hence the conclusion on P-RIOK2 is not convincing.

Our aim in this experiment was to compare the impact of PMA treatment on the phosphorylation levels of different RIOK2 mutants (T481A, S483A, double mutant). In Figure 2C of the original manuscript, the levels of RIOK2 loaded in the two conditions (i.e. not stimulated and PMA stimulated) are very similar for a given mutant, and we therefore assume that our conclusions were valid.
To strengthen our conclusions, we repeated these experiments using T481A and S483A mutants and accurately quantified the data. We replaced the original Figure 2C with a new set of data in the revised version of the manuscript and amended the text accordingly (Page 9, Line 6).

Staining with anti-P RIOK2 lacks controls, how can be sure that the signal is due to the phosphate? Phosphatase treatment?

We fully agree with Reviewer #1 and we did perform an experiment showing that the phosphorylation signal disappears following treatment of the protein extracts with λ-phosphatase. We did not show these data in the original version of the manuscript because of space limitations. We added these data in the supplementary material of the revised version of the manuscript (Supplemental Figure S2B) and amended the text accordingly (Page 8, Line 4)

Why FBS does not lead to ERK staining in HEK293? There are plenty of growth factors in FBS that should lead to ERK phosphorylation. I do not understand this experiment.

We agree with this comment. Addition of serum to starved cells does lead to ERK and RSK phosphorylation but with a much lesser efficiency compared to stimulation by EGF and PMA. ERK phosphorylation is barely visible on the exposure shown in Figure 2D of the original manuscript but RSK-phosphorylation is clearly observed, although the signal is much weaker compared to EGF and PMA treatments. It is common to observe a stronger response with purified PMA and EGF (see Carrière *et al.*, 2011, JBC ; Ray *et al.*, 2013, Oncogene). There are indeed several growth factors in the serum, but the most abundant (Insulin, IGF1, TGF) are present at ng/ml concentrations, while EGF is used at 25 µg/ml in Figure 2D of the original manuscript. Moreover, they are not very strong activators of the Ras/MAPK pathway, and it is also possible that after 20 min of FBS treatment the phosphorylation is in the decreasing phase.

In the present revised version of the manuscript, we included a set of western blots from another experiment showing the same results but in which the effects are more visible (Fig. 2F). We also provided quantifications of RIOK2 phosphorylation and associated statistical analyses (Fig. 2G).

Figure 3. In vitro phosphorylation, if I understood, it relies on a truncated version of RIOK2. Why? Is the folding of the full length protein not permissive to in vitro phosphorylation?

We did not test phosphorylation of the full length RIOK2 protein *in vitro* because RIOK2 has been reported to auto-phosphorylate (Zemp I. *et al.*, 2009, JCB) and we were concerned that this auto-phosphorylation activity of RIOK2 in addition to RSK phosphorylation may render this experiment inconclusive.

HA-RSK3 is less?

It was reported that RSK3 is insoluble when over-expressed (Zhao et al., 1996, JBC), which explains the lower levels of protein recovered in our soluble extract. The information was present in the figure legend but we transferred it to the main text of the result section in the present revised version of the manuscript (Page 10, Line 18).

Figure 4. Immunofluorescence is low mag, difficult to understand.

We agree with Reviewer #1. We modified the FISH experiment figure to show cells with a higher magnification and we provided more details in the text (Page 13, Line 10) to facilitate the understanding of the data.

I really like the experiments with RIOK2 mutants, however I wonder what about protein levels after the knock-in? Given the 18S phenotype overlap between the phenotype of the RIOK2 loss of function with the S483A, testing protein level becomes of the utmost importance.

We checked RIOK2 protein levels and observed that the mutations do not decrease the level of RIOK2. On the contrary, the mutations slightly increase RIOK2 levels. Therefore, we are pretty confident that the phenotypes resulting from expression of RIOK2 mutants do not result from a protein shortage. These data have been added to Figure 4C of the revised version of the manuscript and we amended the text accordingly (Page 13, Line 4).

Figure 5. Low quality IFL.

Our aim in preparing this figure was to show many cells in the different images to show that the effect of our mutation was homogenous at the level of cell populations. The drawback is that cells are small and look blurred. We improved the quality of the figure in this revised version of the manuscript with new images from the same experiment, showing less cells with a higher magnification.

Hard to think that histogram quantitation of nuclear versus cytoplasmic staining are reliable in the absence of fractionation, better quantitation, experiment done in other cell lines and so on.

We provide in this revised version of the manuscript a supplementary figure explaining the procedure we used to quantify the fluorescence data (Supplemental Fig. S7).

In addition, as suggested by Reviewer #1, we confirmed this result by performing cell fractionation assays using eHAP1 cells expressing RIOK2^{WT} or RIOK2^{S483A} allele to monitor the level of the corresponding proteins in the cytoplasmic and nuclear compartments. We included these data in the revised version of the manuscript (Fig. 5C). We also provided quantifications and associated statistical analyses (Fig. 5D). We amended the text accordingly (Page 14, Line 8).

However, very beautiful Fig. 5E perhaps the best of the paper shows also mobility shift driven by S483, thus supporting posttranslational modifications.

We thank Reviewer #1 for this comment. We added the note on the evidence of RIOK2 post-translational modification in the result section (Page 15, Line 3).

Fig. 6. IFL studies are really impossible to interpret.

We improved the quality of the figure with new images from the same experiment, showing less cells with a higher magnification. NOB1 IF data and quantifications have been transferred to the supplemental material (Supplemental Fig. S4A and S4B) to clarify the figure. In addition, we provided more explanations on the principle of this experiment and expected results in the text (Page 16, Line 6).

The effects on RIOK2 release (this figure) and 18S maturation (Fig. 5) are very clear and of great quality.

We thank Reviewer #1 for this comment.

Overall conclusions. The manuscript tends to overinflate the meaning of several experiments. What to me is very clear and interesting is that the the authors provide clear evidence that S483A mutants have a defect in 40S maturation. Whether this is due to MAPK signalling, is only circumstantial. I would suggest to build up on the strong findings and eliminate ambiguous data.

We do not fully agree with this comment of Reviewer #1. If mutation S483A were simply a partial loss of function mutation, this would not be of strong interest for the subject of this manuscript. It would just indicate that S483 is important for RIOK2 function independently of its phosphorylation status. Our data show that the impact of S483 mutation on pre-rRNA processing and other phenotypes is different depending on whether the serine is converted to an alanine (phosphorylation mutant) or to an aspartic acid (phospho-mimetic mutation). These data are a strong indication that what matters is not simply the serine residue by itself, but its phosphorylation status.

Reviewer #1 (Significance (Required)):

The paper deals with an important topic, namely whether a regulation of ribosome maturation exists, and how it is mechanistically regulated. In this context, the analysis of the ERK pathway is highly needed considered that most works deal with effects of the PI3K-mTOR pathway, and the parallel, yet important RAS-ERK pathway, is less understood.

As a final note, we should consider that S6K downstream of mTOR, and ribosomal S6K, downstream of ERK have been considered to share some substrates.

We introduced this information in the revised version of the manuscript (Page 20, Line 21). A related comment has been raised by Reviewer #3 (see below, Caveat #2).

The manuscript is interesting, but several statements given by the authors are rather superficial. An example, listed in the previous section, relates to the linguistic usage of mTOR kinase, instead of detailing whether we are dealing with mTORc1 or mTORc2.

We agree with this comment. Given that the main focus of this manuscript is the regulation by the MAPK pathway, we had chosen to put less emphasis on mTOR in the introduction. However, we added more precise information on mTOR in the revised manuscript to address this comment (Page 4, Line 21 and Page 5, Line 2).

A second gross mistake is the definition of PMA as a stimulator of the ERK pathway. If this is certainly true, this is historically not correct as seminal papers by the group of Parker define this drug as a stimulator of conventional PKC kinases. In short, this paper is a step back in knowledge from the perspective of the literature context.

We are a bit confused by this comment because seminal papers from the Parker group clearly state that PMA activates the MAPK pathway via PKC (Adams and Parker, 1991, FEBS Lett.; Ways et al., 1992, JBC; Whelan et al., 1999, Cell Growth Differ.). We agree, as mentioned earlier by Reviewer #1, that PMA is not specific to MAPK, a comment that has been addressed above.

All people interested to the crosstalk between ribosome maturation and signaling pathways will be certainly read this manuscript.

My expertise is within the ribosome biology and signalling field.

Reviewer #2 (Evidence, reproducibility and clarity (Required)):

There have been mechanistic connections of various signaling pathways to regulation ribosome biogenesis steps including rDNA transcription by RNA polymerase I and III, ribosomal protein transcription, and differential mRNA translation efficiency. However, there is a lacking mechanistic connection of signaling pathways to pre-rRNA processing and maturation steps of ribosome biogenesis. The authors set out to provide a specific example of a direct target of MAPK signaling, RSK that regulates pre-rRNA maturation through the phosphorylation of a ribosome assembly factor (RIOK2), offering for the first time providing mechanistic insight into MAPK regulation of pre-rRNA maturation.

The authors observe slight pre-rRNA processing defects upon the use of RSK inhibitors and RSK depletion. They identified several candidate ribosome assembly and modification factors containing the canonical RSK substrate motif, including the RIOK2 kinase. Phosphorylation at this motif was verified to be specifically phosphorylated by RSK1 and 2 isoforms in cells and in an in-vitro kinase assay. The authors produced RIOK2 knock-in eHAP1 cell lines expressing non-phosphorylatable or phosphomimetic versions of RIOK2, observing slowed cellular proliferation, decreases in global translation, slight pre-rRNA processing abnormalities, but not changes in overall mature 18S rRNA levels. More specifically, the authors defined the inability of RIOK2 to be phosphorylated leads to defects in RIOK2 dissociation from the pre-40S ribosomal subunit in an in-vitro assay, and inability for it to be recycled for reuse in pre-ribosome export from the nucleus to the cytoplasm by immunofluorescence
.

Overall, the authors provide an interesting mechanism of MAPK regulation of a ribosome assembly factor RIOK2. However, they fail to provide the necessary reproducibility, controls, quantification, and consistent results between experiments to support their hypotheses.

Major Comments:

1.The northern blots reported throughout the manuscript are lacking proper reproducibility and quantification. First, the northern blots are lacking a loading control, which is necessary to report fold changes that are being measured across treatments. Please include a proper loading control (i.e. 7SL or U6 RNAs). Additionally, more rigorous analysis of the pre-rRNA precursor levels through ratio analysis of multiple precursors (RAMP) (Wang et al 2014) can be completed to provide a clearer depiction on which precursor(s) are accumulating. It is unclear for the Figure 1 northern blots if there were replicates completed and what the error bars represent in Figure 1B. Please report replicates, so that statistical analysis can be completed on the differences in precursor relative abundance. This need is emphasized by the small changes observed in pre-rRNA levels (less than 2 fold) between conditions.

As mentioned above (Reviewer #1), we systematically applied in the revised version of the manuscript RAMP quantifications to all Northern blot data. These quantifications are shown as separate panels in the figures of the revised manuscript.

Furthermore, we repeated the Northern blot experiments of Figure 1 to obtain biological replicates in two other cell lines in addition to HEK293. All experiments were performed in 3 replicates. We also probed the membranes to detect the 7SL1 RNA as a loading control in all these experiments. We performed RAMP analyses on all these Northern blot experiments and statistical tests to provide more accurate quantifications of the pre-rRNA levels in the different conditions. These data were included in the revised version of the manuscript.

2.The western blots reported throughout the manuscript are lacking proper reproducibility and quantification. For example, the western blots validating RSK1 and RSK2 depletion in Figure 1C lack a proper loading control. Additionally, it is unclear if there are replicates completed and there is lack of statistical analysis to determine if the changes are significant. Please include loading controls, replicates, and quantification of the western blots throughout the manuscript.

We repeated and quantified more accurately the western blot experiments presented in Figures 2A, 2D and 3E. We have included actin levels as loading controls in several figures (Figures 2F, 3A, 3C, 3E, 4C). We also added phosphorylated Rps6 at Ser235/36 to monitor RSK activity in Figures 1A, and 3A.

Regarding Figure 3A, we included another set of western blots showing results using PD and LJH inhibitors of better quality and provided quantifications of phosphorylation of RIOK2 and associated statistical analyses (Fig. 3B). We also provided quantifications and statistical analyses of RIOK2 phosphorylation presented in Figure 3C. We included quantifications of the *in vitro* phosphorylation assays presented in Figures 3F and 3G. All these quantifications and statistical analyses confirmed our initial conclusions.

3.Please report the full bioinformatic analysis of the RSK substrate motif search among human AMFs including other AMFs found in this search. A sorted list format would be valuable for the reader to understand other potential RSK substrates involved in ribosome biogenesis.

We understand the request of Reviewer #2. Providing the full list of AMFs identified in our bioinformatic screen would be valuable for the reader, mostly because it would make clearer that RSK is most likely regulating multiple stages of the pre-ribosome maturation pathway, therefore that RSK inhibition induces pleiotropic defects in ribosome synthesis. However, we are currently working on a more global study of the impact of MAPK regulation on the post-transcriptional steps of ribosome synthesis that we would like to publish in a separate study in a near future.

4.The authors report that RSK inhibition/depletion leads to accumulation of the 30S pre-rRNA, yet mutation of its target site on RIOK2 or RIOK2 depletion leads to an accumulation of the 18S-E pre-rRNA. Additionally, the phosphomimic mutation of RIOK2 leads to an accumulation of 30S, the opposite of the expected result. Please elaborate on this discrepancy in processing defects observed across experiments.

In contrast to RIOK2, which is specifically involved in the late, cytoplasmic stages of the maturation of the pre-40S particles, RSK regulates ribosome biogenesis at multiple levels. Upon activation of the MAPK pathway, RSK activates Pol I transcription in the nucleoli and promotes translation of mRNAs encoding ribosomal proteins and AMFs. In addition, our bioinformatic screen identified several AMFs at different stages of the maturation pathway of both ribosomal subunits as potential targets of RSK. These considerations imply that RSK inhibition is expected to impact ribosome biogenesis at multiple levels (Pol I transcription, availability of RPs and AMFs, export of the pre-ribosomal particles, probably several maturation steps) whereas RIOK2 inactivation more specifically delays 18S-E processing in the cytoplasm. In terms of processing, RSK inhibition induces a significant accumulation of the 30S intermediate. This is another evidence that RSK regulates pre-rRNA processing at several stages. This phenotype might result, as recently described in yeast (Yerlikaya et al., 2016, MCB), from an inhibition of RPS6 phosphorylation which affects its early incorporation into pre-ribosomes, although this has not been demonstrated in human cells. This 30S precursor accumulation affects production of the downstream intermediates and we strongly believe that this precludes accumulation of 18S-E even if the activity of RIOK2 is affected. Given the broad implication of RSK at different stages of ribosome biogenesis, it is biologically relevant to observe that inactivation of RSK does not result in the same processing defects as those resulting from inactivation of RIOK2.

We nevertheless tried to make this point clearer in the revised manuscript. We added in the supplementary material a diagram (Supplemental Fig. S1A) showing all the known and hypothetical targets of ERK and RSK in ribosome synthesis to provide the readers with a global view of the function of RSK in this process and refer to this figure in the introduction and results. In the introduction, we also emphasize more on the multiple aspects of the regulation of ribosome synthesis by ERK and RSK (Page 4, Line 13).

Concerning the phospho-mimetic mutant, it does accumulate slightly the 45S and 30S intermediates contrary to the non-phosphorylatable mutant but this is not totally unexpected. RIOK2 is incorporated into pre-ribosomes in the nucleus, at a stage that remains unclear, and constitutive RIOK2 phosphorylation may interfere with this recruitment and affect processing at an earlier stage. This point has been addressed in the discussion of the revised version of the manuscript (Page 19, Line 6).

Are there similar results for RSK depletion/inhibition and RIOK2 release from the pre-40S and inability to import into the nucleus? If so, this could provide phenotypic consistency between these two proteins in the proposed pathway to further support the hypothesis.

We performed the same experiments as reported in Figure 6C to try to demonstrate a cytoplasmic retention of RIOK2 after leptomycin B treatment upon ERK inhibition (PD treatment). We also performed IF and cell fractionation experiments upon PD treatment. In all cases, we failed to observe the expected result. We strongly believe that we are facing here the same problem as described above for the previous comment of Reviewer #2. ERK and RSK inhibition leads to accumulation of the early, nucleolar 30S intermediate, indicating that the processing pathway is significantly blocked at an early stage preceding formation of the pre-40S particles in which RIOK2 is recruited. This early blockage most likely explains why we do not see the same phenotypes. We discussed this comment in the discussion section of the revised version of the manuscript (Page 19, Line 17).

5.Mature levels of 18S rRNA are not altered in the RIOK2 mutant cell lines. This could be due to compensation in these mutant cell lines since RIOK2 is essential.

We agree with Reviewer #2 that compensation mechanisms may operate to restore mature 18S rRNA levels despite RIOK2 mutation. On the other hand, although RIOK2 is indeed essential, we may expect that the point mutation of S483 only partially affects RIOK2 function and delays the maturation of pre-40S particles but not to a sufficient extent to impact the mature 18S rRNA levels. This has been observed by others (Montellese et al., 2017, NAR; Srivastava et al., 2010, MCB). We added this point in the discussion section of the revised version of the manuscript (Page 20, Line 16).

Please report the mature 18S rRNA levels upon shRNA depletion and RSK inhibitors to provide insight into if this pathway significantly alters mature 18S rRNAs as a mechanism for the altered translation and proliferation observed.

We probed the levels of mature 18S and 28S rRNAs in experiments presented in Figure 1A and did not observed any significant alteration in the presence of PD inhibitor. In contrast, using pulse labeling and chase experiments, we demonstrated that inhibition of the MAPK pathway significantly reduces the amount of mature 18S and 28S produced (Fig. 1C). These data were included in Figure 1 of the revised version of the manuscript and the text was amended accordingly (Page 7, Line 1).

Minor Comments:

1. Figure 1A lower: The authors use an RSK inhibitor LJH685, that does not inhibit RSK phosphorylation S380. Therefore, another verification of RSK inhibition must be used besides RSK-pS380 abundance as for PD184352 inhibition. Please validate the usage of this RSK inhibitor in the experiments by inclusion of quantification of a direct downstream substrate of RSK, such as YB1-pS102 quantification.

We agree with Reviewer #2. In the revised manuscript, the use of LJH685 inhibitor comes later, i.e. in Figure 3. We have probed the membrane with anti-RPS6 and anti-phosho-RPS6 antibodies to show the effect of LJH treatment on RPS6 phosphorylation. These data have been added to Figure 3A and the text has been updated (Page 10, Line 3).

2. Page 7, Lines 8-12: The authors state that RSK knockdown led to increases in the 45S, while the LJH685 treatment led to no changes in 45S levels due to differences in growth conditions. Please elaborate more on how growth conditions would alter 45S pre-rRNA levels. It would be expected that stimulation of the MAPK pathway would increase pre-rRNA transcription compared to steady state growth conditions. However, pre-rRNA processing northern blots are only measuring steady state levels of the precursors. Thus, an rDNA transcription assay would need to be completed to evaluate these differences.

We do observe that PMA treatment of starved cells induces an increase in 45S precursor levels, consistent with an increase in transcription but we agree that northern blot experiments measure the steady-state levels of the intermediates. To address this comment, we performed short pulse labeling with ortho-phosphate to assess in a more dynamic manner synthesis of the 45S precursor. The data confirmed that pre-rRNA synthesis is stimulated upon activation of the MAPK pathway and strengthen our conclusions. These data were included in the revised version of the manuscript (Fig. 1C and 1D).

3. Figure 2C: Please quantify these results to properly evaluate the role of these two phosphorylation sites in MAPK signaling.

We repeated these experiments and quantified the results in the revised version of the manuscript (Fig. 2D and 2E).

4. Please include the RIOK2 pS483 antibody generation methodology used in this study.

We added this information in the Materials and Methods section of the revised version of the manuscript (Page 22, Line 22).

5. In vitro kinase assay methods: Is the recombinant RSK1 the human version of the protein? Please clarify in methods.

Human recombinant RSK1 has been purchased from SignalChem. The information has been added in the revised version of the manuscript (Page 32, Line 9).

6. Figure 4B: Please include statistical analysis of the puromycin incorporation assay.

We performed a statistical analysis of this assay out of 3 replicates. This analysis has been included in the present revised version of the manuscript (Figure 4B).

7. Page 13, Line 18: Please explain why RIOK2 co-IP with NOB1 is important.

We added this explanation in the result section of the revised version of the manuscript (Page 14, Line 23).

8. In vitro dissociation assay: There is no control for pulldown of entire pre-40S particles and not just NOB1 protein. Thus, it is unclear if RIOK2 is dissociating from NOB1 or entire pre-40S particles. Please reference previous literature of the methodology of this experiment if applicable. Additionally, please include controls, such as western blotting of ribosomal proteins or northern blotting of rRNA in the pulldown fraction used.

We agree with Reviewer #2. We have probed the membranes with antibodies detecting LTV1 and ribosomal protein RPS7 to show that the entire pre-40S particle is indeed pulled down. These additional data have been added in Figure 6A of the revised version of the manuscript and the text has been amended accordingly (Page 15, Lines 14 and 23).

9. Page 16, Lines 10-12: The authors state "RSK facilitates the release of RIOK2 and other AMFs", however the only other AMF in this study was NOB1. Please reword appropriately that most likely facilitates release of RIOK2 and other AMFs in a RIOK2 dependent or independent manner if it also phosphorylates other AMFs which possess the motif.

We agree with Reviewer #2 and we changed the text accordingly (Page 17, Line 11) but we did not introduce the hypothesis that RSK may target directly other AMFs of late pre-40S particles which possess the motif because our *in silico* screen did not identify consensus RXRXXS/T motifs in any of these factors.

Reviewer #2 (Significance (Required)):

This manuscript is significant due to the lack of mechanistic connection of cellular signaling pathways to pre-rRNA processing. There have been, for the most part, no mechanistic connection of signaling pathways to pre-rRNA processing regulation and none for direct targets of MAPK signaling (Reviewed in Gaviraghi et al 2019). They provide the groundwork for analysis of MAPK signaling in regulation of an assembly factor and inclusion of their motif analysis could provide RSK signaling targets' regulation of specific steps of ribosome biogenesis that remain to be elucidated.

Although the research delves into a specific mechanism, its audience could be far reaching as it is in the ribosome biogenesis field and MAPK signaling, which have broad implications in cancer and developmental diseases.

Reviewer #3 (Evidence, reproducibility and clarity (Required)):

The authors report that inhibition of MAPK signaling via RSK is associated with modest alterations in the relative abundance of human pre-rRNA species, that are most marked for 30S but also visible for 21S - although not clearly shown for 18S-E.

RIOK2 has two closely spaced sites predicted as RSK targets, one of which was confirmed to be MAPK sensitive and shown to be an RSK substrate in vitro. Substitution of Ser483 with Ala was associated with reduced growth and 18S-E accumulation, consistent with impaired NOB1 cleavage activity. RIOK2-S483A also showed greater pre-ribosome association in vivo and consistent with this, more stable association in vitro and increase cytoplasmic residence. These effects are clear, although the data do not directly demonstrate their linkage to loss of RSK phosphorylation.

The mutations were apparently generated directly in the genome of haploid cells, potentially raising concerns that the introduction of a deleterious mutation might have been accompanied by compensatory mutations elsewhere. However, three cells line gave similar results, mitigating this concern.

Specific comments:

1.To help the reader, the authors should directly discuss why they think the data on MAPK inhibition did not reveal a clearer pre-18S cleavage phenotype, as would have been expected for loss of RIOK2 activity.

This comment is similar to major comment #4 of Reviewer #2.
Please refer to the above response.

2.Fig. S3: The degree of RSK depletion with the siRNAs appears very modest, as are the effects on RIOK2-P. Moreover, the double depletion is not clearly better than single depletions. These data should probably be supported by quantitation or withdrawn.

We agree with Reviewer #3 that the effects shown in this figure are modest but we originally chose to show these data because their further supported the role of RSK in RIOK2 phosphorylation at S483 in complement to Figure 3.
We have withdrawn this figure from the revised version of the manuscript.

3.Fig. 5D: For 18S-E recovery with RIOK2, is the ratio adjusted for the increase in 18S-E abundance in the mutant - ie is recovery increased when adjusted for the increased pre-rRNA abundance?

In these experiments, the tagged versions of RIOK2 WT and S483A have been expressed ectopically from plasmids in cells expressing the endogenous wild-type protein. RIOK2 S483A does not behave as a dominant negative mutant in these conditions and does not induce 18S-E accumulation, as shown in the northern blot analysis of the 18S-E levels in the cell lysates (lower panel). This information is indicated in the revised version of the manuscript (Page 14, Line 20).

Reviewer #3 (Significance (Required)):

Overall, the analyses on the phenotype of RIOK2-S483A, and the demonstration that this site is an RSK target, appear convincing.

Caveats are

1) the phenotype seen on inhibition of RSK, would not have implicated RIOK2 as the obvious candidate for the factor responsible for the observed processing defects;

We agree with this comment, which has also been raised by Reviewer #2 (Major comment #4). We provide several evidence in the manuscript that RSK phosphorylates RIOK2 on S483 in vivo and in vitro (Figure 3). However, as explained above in response to Reviewer #2, we cannot correlate the in vivo phenotypes resulting from RSK or RIOK2 inactivation for biological reasons.

As mentioned in the introduction, RSK regulates multiple substrates at different stages of ribosome biogenesis (Translation of RPs and AMFs, Pol I transcription, pre-ribosome maturation and export), whereas RIOK2 is specifically implicated in the cytoplasmic maturation of pre-40S particles. Inactivation of RSK is therefore expected to induce pleiotropic defects in ribosome biogenesis, and in particular early defects (Reduced Pol I transcription, 30S precursor accumulation) that preclude observation of the expected phenotype linked to RIOK2 inactivation, i.e. 18S-E accumulation.

We nevertheless tried to clarify this point as described in the response to Reviewer #2, major comment #4.

2) the RIOK2-S483A phenotype is not demonstrated to be RSK dependent. This raises the possibility that, although RSK can phosphorylate S483, the effects of the mutation are not due to the loss of this modification.

As mentioned by Reviewer #3, our data show that RSK can phosphorylate RIOK2 S483 *in vitro* and *in vivo* (Figure 3). We believe that Figure 4C strongly suggests that the accumulation of the 18S-E in cells expressing RIOK2 S483A mutant is due to the loss of S483 phosphorylation, since mutation of S483 to an aspartic acid (S483D), generally considered as a mutation mimicking a phosphorylated serine, does not affect 18S-E maturation. However, although our manuscript provides many lines of evidence identifying RSK as the kinase responsible for RIOK2 phosphorylation at S483, we cannot formally exclude that other AGC kinases involved in growth and proliferation, such as S6K or Akt, may also be involved redundantly or alternatively. Our data presented in Figure 3A showing that treatment of cells with the RSK inhibitors LJH decrease RIOK2 phosphorylation at S483 support a specific role of RSK.

We developed this point in the discussion section (Page 18, Line 24).

With these provisos, the work is technically good and will be of considerable interest to the field. The post-transcriptional regulation of ribosome synthesis is increasingly recognized a significant topic.
